# Development, psychometric evaluation, and initial feasibility assessment of a symptom tracker for use by patients with heart failure (HFaST)

**DOI:** 10.1186/s41687-019-0113-6

**Published:** 2019-05-02

**Authors:** Eldrin F. Lewis, Theresa M. Coles, Sandy Lewis, Lauren M. Nelson, Amy Barrett, Carla DeMuro Romano, Donald E. Stull, Stuart J. Turner, Chunlan G. Chang

**Affiliations:** 1 Brigham and Women’s Hospital, Boston, MA USA; 2 RTI Health Solutions, 3040 East Cornwallis Road, PO Box 12194, Research Triangle Park, NC 27709-2194 USA; 3 Novartis Pharmaceuticals Corporation, East Hanover, NJ USA

**Keywords:** HFaST, Heart failure, Communication, Symptom tracker, Psychometric testing, Qualitative approaches, Instrument development

## Abstract

**Background:**

This study aimed to develop and provide a psychometric and feasibility pilot evaluation of the Heart Failure (HF) Symptom Tracker (HFaST), a new patient-reported tool designed to facilitate communication between patients and health care providers (HCPs) in routine clinical care. The HFaST enables patients to identify worsening HF symptoms, with a long-term goal of preventing hospitalizations or emergency room visits.

**Methods:**

The HFaST was developed drawing on evidence from the literature, qualitatively with cognitive interviews (12 patient/caregiver and 8 HCPs), and evaluated quantitatively (psychometric, feasibility assessment). The HFaST was administered for 7 consecutive days to 100 individuals diagnosed with HF during a multisite, non-interventional US pilot study. Health care providers then completed a survey assessing the feasibility and importance of the HFaST in clinical practice.

Qualitative development included a literature review and cognitive interviews with patients, caregivers, and HCPs. The psychometric properties of the HFaST were evaluated using classical test theory methods. Descriptive statistics provided insight into HCPs’ perceptions of the feasibility of using the HFaST in clinical practice.

**Results:**

A preliminary set of 40 items was developed for the symptom tracker and iteratively reduced to 10 items based on the qualitative phase. Test-retest reliability (weighted kappa 0.71–0.97), discriminating validity, and construct validity of the HFaST were acceptable. HCPs rated the HFaST as a good (70%) or excellent (30%) means of tracking HF symptoms. Six HFaST items were ultimately retained, covering concepts of fatigue, shortness of breath (3 items), swelling, and rapid weight gain.

**Conclusions:**

The 6-item HFaST is an easy-to-use tool designed to raise patients’ awareness of HF symptoms and facilitate communication with HCPs. Future research should evaluate HFaST implementation in clinical practice and effectiveness as an intervention to potentially prevent hospitalizations and emergency room visits.

**Electronic supplementary material:**

The online version of this article (10.1186/s41687-019-0113-6) contains supplementary material, which is available to authorized users.

## Background

Heart failure (HF) affects approximately 26 million people worldwide [1]. Heart failure is the primary diagnosis in more than 1 million hospitalizations in the United States (US) annually [2] and accounts for the largest proportion of all 30-day rehospitalizations for the US Medicare population [3]. Many HF-related hospitalizations occur because patients or caregivers fail to recognize when symptoms first progress and seek intervention [4]. As policymakers aim to reduce HF-related hospitalizations [5], a brief and easy-to-use communication tool is needed to improve patients’ recognition of worsening HF symptoms.

In clinical practice, patient-reported outcome (PRO) measures detect problems that may otherwise be overlooked, facilitate symptom monitoring and communication between health care providers (HCPs) and patients, and improve patient satisfaction with care [6–8]. Clinical measures can be less predictive of HF-related hospitalizations than patient reported outcome (PRO) measures [9], suggesting that HF-specific PRO measures could be beneficial in improving patient outcomes.

Several patient-completed, HF-specific instruments have been developed to assess quality of life, symptoms, and functioning [10, 11]. However, most available HF measures are not ideal for administration in clinical practice due to their length and scoring complexity. Further, most employ lengthy recall periods, introducing the potential for recall bias and hindering patients’ prompt identification and communication of rapidly worsening HF symptoms that may require intervention.

The HF Symptom Tracker (HFaST) was designed to be integrated in routine clinical care in outpatient settings and increase patient awareness of important increases in HF symptom severity that may require intervention. Importantly, the focus of this work is on the rigorous development and initial testing. Operationalization of the tool in outpatient settings is an important next step. The objectives of this pilot study were to integrate input from patients, caregivers, and HCPs in the development of a brief, self-reported measure (the HFaST); evaluate the psychometric properties of the HFaST; assess HCPs’ perceptions of the HFaST feasibility in clinical practice; and optimize the items included in the final HFaST.

## Methods

**Fig. 1:**
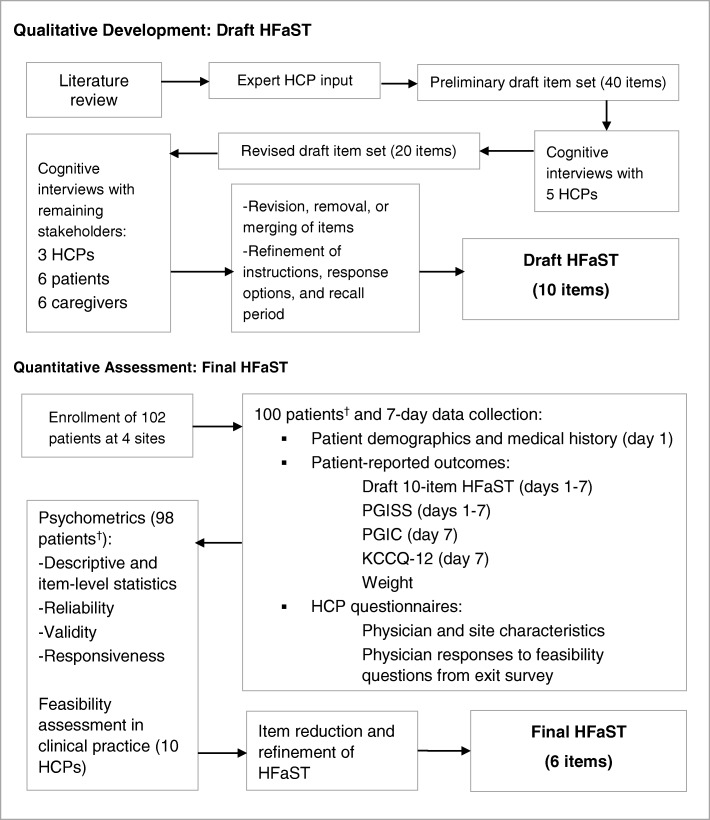
HFaST Development Process. *Two (2) patients were lost to follow-up. † Of the 100 patients who began the study, 98 participated in at least 4 HFaST study days. HCP = health care provider; HFaST = Heart Failure Symptom Tracker; KCCQ-12 = Kansas City Cardiomyopathy Questionnaire; PGIC = Patient Global Impression of Change; PGISS = Patient Global Impression of Symptom Severity

Figure 1 illustrates the qualitative and quantitative evaluation process for the HFaST.

### Participants

Patient inclusion and exclusion criteria were consistent for the qualitative and quantitative assessments: Adults with a diagnosis of HF who could read and understand English and who had previously experienced HF-related shortness of breath, fatigue, or swelling were eligible. Patients with a history of any severe lung disease and patients undergoing cancer-directed chemotherapy were ineligible. The study was conducted in outpatient settings. All participants received reimbursement for study participation (HCPs at Fair Market Value and patients/caregivers $100).

Caregivers (qualitative assessment only) were individuals who lived with, spent four or more hours per day with, and provided care for an individual with HF. Eligible HCPs were physicians or nurses who routinely treated patients with HF.

Health care providers were included in the qualitative and quantitative studies. For the quantitative study, up to three HCPs from each of the 10 sites participated in the HCP exit survey.

### Qualitative development methods

The patient-reported HFaST was developed through a rigorous process that included conducting a targeted literature review (conducted March 28, 2015; articles indexed in PubMed in the past 10 years), obtaining expert clinician input, and conducting qualitative interviews with individuals diagnosed with HF, caregivers, and HCPs. The combined concept elicitation/cognitive debriefing approach allowed for both spontaneous capture of important symptoms as well as an opportunity for participants to highlight any possible missing items. Following introductions among interviewers and participants, an explanation of the study and objectives, and concept elicitation, patients and caregivers participated in cognitive debriefing to evaluate the questionnaire instructions, recall period, and response choices. The methods presented in Fig. 1 are described in more detail here. Convenience sampling for patients and caregivers was utilized.

Employing standard item development principles [12], RTI-HS created a preliminary, comprehensive 40-item version of the HFaST based on the literature review findings and expert clinician input. RTI-HS conducted cognitive interviews [13] with 8 HCPs including nurses with extensive cardiac care experience (*n* = 2), primary care physicians (family medicine, internal medicine, or general practice physicians) who treat patients with HF (*n* = 4), and cardiologists (n = 2). These interviews provided insight into which HF symptoms are most relevant for providing a clear representation of the patient’s health status related to HF, the ideal recall period and frequency of assessment, and the response choices that provide the most useful information for HCPs and for patients monitoring their HF symptoms.

Following conduct of just over half (*n* = 5) of the HCP interviews (i.e., 2 nurses and 3 primary care physicians), the HFaST was refined and the total number of draft items was reduced from 40 to 20. This refined item set was further tested with the remaining stakeholders (i.e., 2 cardiologists, 1 primary care physician, 6 patients, and 6 caregivers).

Trained medical recruiters invited HCPs by e-mail to participate in the qualitative study, and a qualitative research firm in Raleigh, North Carolina invited patients and caregivers by e-mail to participate.

During telephone interviews, HCPs were asked to describe HF signs and symptoms that were most clinically relevant or indicative of the need to seek medical attention. Health care providers also provided input on the relevance and clinical meaningfulness of draft HFaST items and evaluated the instructions, response options, and recall period.

### Qualitative interview conventions

Four trained and experienced qualitative researchers conducted face-to-face, individual concept elicitation interviews with patients or caregivers (one interviewee and two interviewers). Patient and caregiver interviews took place in a private room at a qualitative research facility.

All interviewers followed semi-structured interview guides designed to incorporate concept elicitation through open-ended questions and targeted probes as well as a “think aloud” technique to facilitate cognitive debriefing. All patient and caregiver participants provided informed consent (consent for HCP interviews was not required because no personal medical information was shared). Each interview lasted approximately 1 h. Interviews were audio recorded and transcribed to support content analysis [14], which was facilitated by the interviewers’ field notes.

### Quantitative assessment methods

#### Design and population

The 10-item draft HFaST was evaluated during a 6-site, non-interventional pilot study conducted in March–October 2016. The enrollment target was approximately 100 patients. Recruitment used a nonprobability sampling scheme that prioritized representativeness across the HF severity spectrum using New York Heart Association (NYHA) classifications. To limit the proportion of asymptomatic patients, staff at each participating site were instructed to enroll at least eight patients reporting at least one key HF symptom during screening (e.g., shortness of breath; swelling in feet, ankles, legs or abdomen; heart palpitations; rapid weight gain of 2 pounds or more within 24 h). For clinic-based sites, trained study coordinators performed chart reviews of patients with a HF diagnosis followed by a phone interview to determine eligibility using the same criteria. For interested patients, staff obtained informed consent and conducted patient training on how to access study materials and when to complete questionnaires.

For patient recruitment sites (*n* = 2), patients were recruited by study site staff via direct-mail invitation letter or e-mail. Patients were prescreened using a standard script, with confirmation of clinical eligibility from the patient’s cardiologist using the same criteria used at clinic-based sites. Informed consent was obtained from interested, eligible patients who were trained prior to study participation.

Clinic-based participants were given the option of completing the study assessments electronically or via paper forms. Patients who were enrolled at patient recruitment facilities were administered the study questionnaires via web only. The pilot study period was 7 consecutive days for each patient, during which all patients received usual care.

#### Instruments

The following measures were administered during the quantitative study:

- Recruitment screening tool

The recruitment screening questionnaire was used to collect patient demographics and key medical history information.

- HFaST

The draft HFaST tool included 10 items (Table 1). For 9 of the 10 draft HFaST items, an 8-point response scale was used to evaluate the status of HF symptoms over the previous 24 h compared to usual (0 [“Did not experience in the past 24 hours”]; 1 [“Much better than usual”]–7 [“Much worse than usual”]). A dichotomous (yes/no) response was used for HFaST Item 10 (weight gain). The HFaST was administered to patients daily for 7 consecutive study days.

- Kansas City cardiomyopathy questionnaire (KCCQ-12)

**Table 1:** HFaST Item-level Response Distributions for Study Day 4

HFaST Score	Did not experience in the past 24 h^a^	Much better than usual^a^	Somewhat better than usual^a^	Slightly better than usual^a^	About the same as usual^a^	Slightly worse than usual^a^	Somewhat worse than usual^a^	Much worse than usual^a^	Missing (%)
HFaST 1 Fatigue or low energy level when performing everyday activities	22 (22.9)	3 (3.1)	8 (8.3)	8 (8.3)	34 (35.4)	16 (16.7)	4 (4.2)	1 (1.0)	2 (2.0)
HFaST 2 Fatigue or low energy level even while sitting or lying down	32 (33.7)	5 (5.3)	1 (1.1)	10 (10.5)	29 (30.5)	13 (13.7)	5 (5.3)	0 (0)	3 (3.1)
HFaST 3 Shortness of breath when performing everyday activities	31 (32.6)	3 (3.2)	7 (7.4)	5 (5.3)	29 (30.5)	16 (16.8)	4 (4.2)	0 (0)	3 (3.1)
HFaST 4 Shortness of breath at rest	41 (42.3)	5 (5.2)	5 (5.2)	10 (10.3)	27 (27.8)	9 (9.3)	0 (0)	0 (0)	1 (1.0)
HFaST 5 Shortness of breath while lying down or reclining (for example, needing to add pillows or move to a recliner to sleep)	51 (52.6)	3 (3.1)	5 (5.2)	5 (5.2)	28 (28.9)	4 (4.1)	0 (0)	1 (1.0)	1 (1.0)
HFaST 6 Sudden attacks of shortness of breath that wake you from sleeping	64 (68.1)	4 (4.3)	4 (4.3)	4 (4.3)	12 (12.8)	6 (6.4)	0 (0)	0 (0)	4 (4.1)
HFaST 7 Cough	41 (42.7)	8 (8.3)	7 (7.3)	10 (10.4)	23 (24.0)	7 (7.3)	0 (0)	0 (0)	2 (2.0)
HFaST 8 Swelling of feet, ankles, legs, or abdomen; shoes or waistband feeling tight	41 (42.3)	6 (6.2)	5 (5.2)	8 (8.2)	23 (23.7)	10 (10.3)	3 (3.1)	1 (1.0)	1 (1.0)
HFaST 9 Heart palpitations—rapid, fluttering, or pounding heartbeat	59 (60.8)	4 (4.1)	2 (2.1)	2 (2.1)	21 (21.6)	7 (7.2)	1 (1.0)	1 (1.0)	1 (1.0)
HFaST 10 Gained more than 2 pounds during the past 24 h or more than 5 pounds during the past 72 h	No = 79 (81.4)	Yes = 18 (18.6)							

*HFaST* Heart Failure Symptom Tracker

Note: HFaST values are as follows: 0 = Did not experience in the past 24 h, 1 = Much better than usual, 2 = Somewhat better than usual, 3 = Slightly better than usual, 4 = About the same as usual, 5 = Slightly worse than usual, 6 = Somewhat worse than usual, 7 = Much worse than usual

^a^ Percent calculated out of non-missing responses

The KCCQ-12 is a 12-item, patient-reported, multidimensional instrument that quantifies HF-related physical function, symptoms, social function, and quality of life [15]. Subscale and composite scores range from 0 to 100, with higher scores reflecting better health status. The KCCQ-12 was administered on day 7 and has a recall period of the “past 2 weeks.” The minimal clinically important difference for the KCCQ-12 is ≈3 to 5 points.

- *Patient global impression of change (PGIC)*

The PGIC, a single item [16], was completed on day 7 and assessed change in HF symptom severity from the patient’s perspective since the beginning of the study. The PGIC employs a 7-point graded response scale (1 = Very much improved, 2 = Much improved, 3 = Minimally improved, 4 = No change, 5 = Minimally worse, 6 = Much worse, and 7 = Very much worse).

- *Weight and symptom severity*

Additional patient-reported assessments included a daily weight report and five daily HF symptom severity items (global severity on the Patient Global Impression of Symptom Severity [PGISS] covering fatigue, shortness of breath, cough, swelling, and palpitations). The PGISS items were rated on a 5-point scale from 0 (“None”) to 5 (“Very severe”).

- *HCP exit questionnaire*

A paper-based exit questionnaire was administered to HCPs in order to assess the feasibility and importance of the HFaST in clinical practice (Additional file 1: Figure S1).

#### Psychometric evaluation

Psychometric properties of the draft HFaST were evaluated and descriptive statistics were computed summarizing HCP-reported acceptability and feasibility of the measure. Only patients who responded to the HFaST for at least 4 of 7 days were included in the analyses.

##### Distributional characteristics

Performance of the draft HFaST items was evaluated using descriptive statistics and item frequency distributions across the 7-day period to provide insight into potential response biases.

##### Structure and reliability

Inter-item polychoric correlations were computed between pairs of HFaST items to examine patterns of associations. Test-retest reliability was evaluated using item-level weighted kappa statistics. Because the HFaST retrospectively captures symptom changes over the previous 24 h compared to usual, change in HFaST scores from 2 consecutive days were compared with a stable subsample of patients in which no change was reported on PGISS items for 3 consecutive days [17]. “Test” data were HFaST item-level scores on the first day; “retest” data were HFaST item-level scores on the next day. Kappa statistics above 0.80 were considered almost perfect agreement [18].

##### Validity and responsiveness

Construct validity of the HFaST was assessed. Frequency distributions of HFaST scores were computed for patients who responded “Never over the past 2 weeks” on select KCCQ-12 items. It was anticipated that more patients who did not experience a symptom over the previous 2 weeks based on the KCCQ-12 would also report “Did not experience in the past 24 hours” on a corresponding HFaST item. Fatigue and shortness of breath were assessed analogously.

Responsiveness was assessed by grouping patients into one of three PGISS categories of change (improvement, no change, or worsening) for subsequent days (e.g., day 1 to day 2, day 2 to day 3), and descriptive statistics for HFaST scores on the second day of each change pair were calculated for items measuring matching concepts. Patients who did not report having experienced an HFaST symptom in the previous 24 h were removed from the analysis for that item and that day. HFaST scores were expected to show an increasing trend, with lower average HFaST scores for patients categorized as improving on PGISS and higher HFaST scores for patients categorized as worsening on the matching PGISS item.

Known-groups analyses comparing subgroups of interest were conducted to evaluate the discriminating ability of the HFaST. Patients were categorized into two groups based on the PGIC (improvement/no change versus worsening). It was hypothesized that patients who endorsed PGIC response choices describing worsening symptoms would yield higher (worse) mean HFaST item scores averaged over the week than patients who reported no change or improvement on the PGIC.

#### Feasibility and acceptability

Descriptive statistics were computed to summarize responses to the HCP exit survey, which evaluated feasibility and acceptability of the HFaST.

#### Item reduction

Ten draft items were evaluated for inclusion in the final HFaST based on qualitative evidence, the HCP exit survey, and the psychometric results. The goal was to preserve face and content validity but remove redundant or poor-performing items.

### Ethical considerations

All study procedures were approved by the Office of Research Protection and Ethics at RTI International. Informed consent was obtained for all study participants.

## Results

### Qualitative development of the HFaST

Of the HCP interview participants, two were cardiac care nurses, four were primary care physicians, and two were cardiologists (50% female; mean 26.0 years in practice [range, 15–38]). The interviews also included six patients (mean age, 58.2 years [range, 38–67]; 66.7% female; 50% college/advanced degree; 83.3% had an emergency room [ER] visit) and six caregivers (mean age, 50.2 years [range, 32–60]; 83.3% female; 66.7% college/advanced degree). The varied sample ensured representative feedback from relevant stakeholders.

#### Key concepts

##### Item set/instructions

Based on the literature review and HCP feedback, a preliminary set of 40 items was developed (Additional file 1: Figure S2) and piloted with five HCPs to identify those items most relevant for inclusion. Most of the HCPs felt that frequent monitoring of symptoms by patients was needed.

The HCPs found the instructions clear and approved of the emphasis on patients reporting “any new or worsening symptoms” and thinking about “changes in your symptoms.” One of the cardiac nurses pointed out that some HF symptoms may not be appropriate for patients to track (e.g., confusion, fainting, restlessness, dizziness, and lightheadedness), as they require “clinical determination” and would be difficult for patients to self-report.

##### Symptoms/impacts

All of the HCPs stressed the importance of understanding the impact of fatigue and/or shortness of breath on patients’ ability to do daily activities, referred to as “activity tolerance.” Several HCPs suggested also assessing fatigue and shortness of breath while at rest or while sitting, in addition to fatigue and shortness of breath associated with activity. Four of five HCPs said that swelling in different locations (abdominal or lower extremity) did not need to be assessed separately, and therefore only one item on swelling may be needed. Two HCPs stated that specifying the type of cough was less important and further noted that patients might not be familiar with the descriptors “dry (nonproductive)” and “wet-sounding.” HCPs reported that some items (e.g., wheezing, slow/ irregular heart rate, nausea, vomiting, diarrhea, and discolored skin) were less clinically meaningful for assessing worsening HF signs and symptoms.

##### Revisions

Based on HCP feedback, the HFaST was reduced to 20 items (see Additional file 1: Table S1 for a summary of the 40- to 20- item versions). The 20 items presented in Additional file 1: Table S1 were then evaluated for inclusion in combined concept elicitation/cognitive debriefing interviews with three additional HCPs, six patients, and six caregivers.

#### Stakeholder review

##### Symptoms/impacts

First, stakeholders provided feedback on the 20 -item version (item content presented in Additional file 1: Table S1) via in-depth cognitive interviews. Patients understood the term “everyday activities” and provided examples such as playing golf, cooking, housekeeping, and grocery shopping. Generally, patients found the items addressing tiredness, fatigue, low energy level, and low energy level when performing everyday activities to be very similar to one another but distinct from the item asking about generalized weakness. Among the patients who expressed a preference among these items, two preferred tiredness, and one preferred low energy level. No patient preferred generalized weakness or low energy level when performing everyday activities. The item assessing generalized weakness was removed from subsequent versions of the HFaST. All of the patients were familiar with the term “shortness of breath.” Patients provided examples of activities that would or would not exacerbate shortness of breath: cooking (no shortness of breath), walking to get mail (shortness of breath), walking from car (shortness of breath), being outside when the temperature was high (shortness of breath). The phrase “Shortness of breath that requires use of additional pillows” was interpreted inconsistently by patient participants. Some thought that the “additional pillows” were pillows in excess of the number that they usually used, and some thought they were pillows other than the one pillow that would be considered “normal.” Thus, item 8 was reworded as “Shortness of breath while lying down or reclining (for example, needing to add pillows or move to a recliner to sleep).” Two patients said that they wake up from sleeping but were not sure if this was because of shortness of breath or because they needed to go to the bathroom. Based on this feedback, the sleeping item (which was intended to capture occurrences of paroxysmal nocturnal dyspnea) was reworded as “Sudden attacks of shortness of breath that wake you from sleeping.”

##### Item revisions

Generally, patients indicated that dizziness/lightheadedness or feeling confused rarely applied to them, but that they would be concerned if they experienced any of these symptoms related to HF. Three patients said they would sometimes feel lightheaded while bending over, standing from sitting, or after overdoing activities. One patient said that she sometimes felt confused, and another said that she had trouble differentiating confusion due to HF from confusion due to medications or age. Both of these items (dizziness and lightheadedness) were deleted following interviews with all of the stakeholder types who reviewed the 20-item symptom tracker. This decision was made on the advice of the HCPs and was supported by the fact that patients rarely experienced these symptoms.

Generally, patients did not associate wet or dry cough with HF (instead, they attributed cough to other conditions such as flu cold, thirst), and most did not understand one or both of the two descriptions of cough (i.e., dry (nonproductive cough), wet-sounding cough). Because patients were not able to interpret the cough descriptors or rate their experience with different types of cough, the two items assessing types of cough were combined into one item assessing cough with no further description.

Patients reported swelling in different areas of their bodies. Two patients indicated that they experienced abdominal swelling, three experienced swelling in their feet or ankles, and two experienced swelling in their legs. Therefore, one combined question about swelling (Swelling of feet, ankles, legs, or abdomen; shoes or waistband feeling tight) was included in the 10-item HFaST.

Because no amount of weight gain was specified in the item to clarify what was meant by “rapid weight gain,” patients found this item difficult to interpret. In the 10-item HFaST, this item was changed to a yes/no question: “Have you gained more than 2 pounds during the past 24 hours or more than 5 pounds during the past 72 hours?” HCPs supported this revision and suggested the threshold amounts of weight gain over brief time periods that should trigger patients and caregivers to contact an HCP.

Most patients either did not associate the symptoms described in the eating-related items (feeling full sooner than is typical, loss of appetite) with HF, or they did not experience the symptoms. Given the difficulty of interpreting these items and the nonspecific nature of these items, these two items were deleted.

Patients seemed to have an understanding of rapid, fluttering, and pounding as they related to heartbeat; this item was retained with no changes. Generally, patients did not endorse feeling cold or numbness/tingling as HF symptoms; based on this feedback, as well as HCP input that the etiology of these symptoms may not be exclusive to HF, these items were removed from the HFaST.

##### Response options

Response choices were also evaluated. Patients were asked to provide their feedback on an 11-point numerical rating scale (NRS) and a 5-point NRS evaluating current status, both with descriptive anchors, as well as a 5-point verbal rating scale. With the 11-point NRS, all patients understood the anchors (“no symptom” and “worst imaginable”), but a number of patients reported that “worst imaginable” was too strong or dramatic. Patients expressed difficulty differentiating the meaning between adjacent numbers on the 11-point scale. Almost all noted that it was difficult to choose a response using the 11-point NRS. Patients preferred a 5-point response scale and thought responses on a 5-point scale would be more accurate.

It became clear as patients and caregivers described their thought processes in selecting responses that they were aware of the patient’s usual status related to HF symptoms and some took this into consideration in choosing a response. For example, a patient who had reported experiencing “moderate” fatigue on most days selected 1 on the 11-point NRS because his fatigue had not been worse than usual in the past 24 h. Some caregivers interpreted the 11-point NRS rating scale as an absolute assessment of symptom severity during the past 24 h, and others interpreted it as an assessment of symptom severity during the past 24 h versus the patient’s normal status.

A cardiologist noted that for clinicians, understanding the change in symptoms (more severe symptom or a new symptom) is more important than current symptom status. He added, “it is the provider’s job to establish the diagnosis and the baseline and then a tool like this can help with follow-up, but tracking symptom change is the most important goal of this type of questionnaire.” He went on to say that patients “may always feel fatigued or have mild or moderate edema, and they may always give these symptoms high ratings.” Knowing when HF patients believed their symptoms were “worse than usual” or “much worse than usual” was key to effective communication for this provider. Based on findings from all three shareholder groups, the response choices were changed from rating symptom severity to comparing the symptom during the past 24 h to “what you usually experience.”

##### Recall period

Finally, the recall period was evaluated. Most patients preferred a short (24-h) recall period (4 patients) to a 7-day recall period (2 patients). Of the patients who preferred a longer recall period, they expressed that it took at least a week, or possibly as much as a month, to experience a change in their heart failure symptoms. Patients who preferred a shorter recall period indicated that some HF symptoms could change within hours or days, or that they could not remember the severity of HF symptoms from more than 3 days ago. Caregivers generally agreed that the 24-h recall period was appropriate for capturing change in heart failure symptoms. One caregiver explained “a lot can go on in 24 hours.” All HCPs in this round agreed that the 24-h recall period was appropriate. Ultimately, the 24-h recall period was selected based on the need for accurate patient recall as well as to allow identification of deterioration that can occur rapidly.

Based on the combined feedback, which was aligned across the stakeholder groups, 10 items were retained (item content presented in Table 1), a 24-h recall period was employed to capture rapidly changing HF symptoms, and a response-choice format was selected to allow patients to communicate HF symptom changes relative to a usual state.

### Quantitative assessment

#### Response rate and description of participants

Patients were recruited from four clinics and two patient recruitment facilities. Of the 102 patients enrolled in the quantitative study, 2 patients were lost to follow-up and 98 patients participated in at least 4 HFaST study days (i.e., 98% participation in HFaST for ≥4 study days).

Patients’ mean age was 58.8 years (standard deviation [SD], 13.2; range, 24–87) and approximately half of the patients were white (46.9%). Almost 30% of the sample was newly diagnosed with HF (27.5% within the last year). The sample included patients from all four NYHA classifications [19]: class I, 13.3%; class II, 54.1%; class III, 25.5%; class IV, 7.1%. Of patients reporting comorbidities, the most prevalent were hypertension (72.3%) and irregular heartbeat (48.9%). Almost all patients had been seen at the ER for HF symptoms in their lifetime (78.6%). There was a patient preference for paper survey administration, with 57.1% of the sample completing paper-based questionnaires. Some sites provided only a web-based option. Therefore, patients’ preference for paper-based HFaST surveys may be higher than 57.1%.

Ten HCPs completed the exit questionnaire. All respondents were engaged with the research participants and provided routine care to patients in their practice. More than half of the HCP sample (60%) had been practicing for 10+ years, and 33.3% were associated with a hospital-based clinic. A majority (70.0%) used PRO measures in clinical practice.

#### Psychometric evaluation

Although the results patterns were reviewed and are reported for all days, day 4 was selected as the primary study day for demonstration because it represented a midpoint for data collection, and day 4 results were generally representative of other study days.

##### Item performance

Day 4 HFaST item-level response distributions presented in Table 1 (Additional file 1: Table S2 for all days) demonstrate an acceptable range, with no apparent floor or ceiling effects across the 7 days. However, over 50% of the sample reported not experiencing a symptom or no change from usual, indicating a fairly asymptomatic sample. The least prevalent symptoms were “shortness of breath at rest” (HFaST Item 4), “shortness of breath while lying down or reclining” (HFaST Item 5), “sudden attacks of shortness of breath that wake patient” (HFaST Item 6), “heart palpitations” (HFaST Item 9), and “gaining more than 2 pounds in the past 24 hours or 5 pounds in the past 72 hours” (HFaST Item 10); 40% or more of patients reported “did not experience” for these items on all 7 study days.

Mean KCCQ-12 subscale scores ranged from 46.6 (SD, 27.5) in quality of life to 58.8 (SD, 26.9) in symptom frequency in the past 2 weeks (Additional file 1: Table S3). Less than 2% of participants described their HF symptoms as “Very Severe” on the PGISS items (Additional file 1: Table S4). The lowest (least severe) mean severity scores were associated with PGISS Heart Palpitations, with average severity scores between “None” and “Mild” (mean, 0.60–0.75); PGISS Fatigue yielded the highest mean severity scores, between “Mild” and “Moderate” (mean, 1.21–1.34) (Additional file 1: Table S4). Based on change in PGISS, patients reported very little change in HF symptoms, with mean change scores ranging between − 0.1 and 0.1 and median change scores at 0 for all five PGISS items (Additional file 1: Table S5). Furthermore, 42% of patients reported “No change” on the PGIC. Average weight was stable, ranging from a low on day 6 of 220.8 pounds (SD, 58.7) to a high on day 1 of 221.5 (SD, 58.8) (Additional file 1: Table S6).

##### Structure

Inter-item correlations are presented for study day 4 (Table 2). Correlations on day 7 [0.22 ≤ |r| ≤ 0.92]) were slightly stronger than those on day 1 (0.09 ≤ |r| ≤ 0.89 [Additional file 1: Table S7]). Pairs of strong (r > 0.50) correlations, potentially indicating redundancy, were exhibited at all study days between the following items: “fatigue or low energy level while performing everyday activities” (HFaST Item 1) and “fatigue or low energy level even while sitting or lying down” (HFaST Item 2) (0.74 ≤ r ≤ 0.90); “fatigue or low energy level even while sitting or lying down” (HFaST Item 2) and “shortness of breath while performing everyday activities” (HFaST Item 3) (0.56 ≤ r ≤ 0.79); “shortness of breath while performing everyday activities” (HFaST Item 3) and “shortness of breath at rest” (HFaST Item 4) (0.69 ≤ r ≤ 0.85); “shortness of breath at rest” (HFaST Item 4) and “shortness of breath while lying down or reclining” (HFaST Item 5) (0.84 ≤ r ≤ 0.91); and “shortness of breath while lying down or reclining” (HFaST Item 5) and “sudden attacks of shortness of breath that wake you from sleeping” (HFaST Item 6) (0.74 ≤ r ≤ 0.92). Generally, inter-item correlations with “gaining more than 2 pounds in the past 24 hours or 5 pounds in the past 72 hours” (HFaST Item 10) were weaker than correlations among HFaST items 1 through 9.

**Table 2:** Inter-item Correlations on Study Day 4 (*n* = 92 to 97)

HFaST Item	1	2	3	4	5	6	7	8	9	10
HFaST 1 Fatigue or low energy level when performing everyday activities	1									
HFaST 2 Fatigue or low energy level even while sitting or lying down	0.82	1								
HFaST 3 Shortness of breath when performing everyday activities	0.62	0.67	1							
HFaST 4 Shortness of breath at rest	0.53	0.68	0.75	1						
HFaST 5 Shortness of breath while lying down or reclining (for example, needing to add pillows or move to a recliner to sleep)	0.56	0.68	0.74	0.91	1					
HFaST 6 Sudden attacks of shortness of breath that wake you from sleeping	0.45	0.49	0.52	0.74	0.81	1				
HFaST 7 Cough	0.46	0.33	0.56	0.58	0.57	0.44	1			
HFaST 8 Swelling of feet, ankles, legs, or abdomen; shoes or waistband feeling tight	0.62	0.48	0.53	0.60	0.60	0.56	0.51	1		
HFaST 9 Heart palpitations—rapid, fluttering, or pounding heartbeat	0.54	0.69	0.64	0.67	0.69	0.53	0.53	0.45	1	
HFaST 10 Have you gained more than 2 pounds during the past 24 h or more than 5 pounds during the past 72 h?	−0.05	− 0.05	−0.27	− 0.12	−0.13	− 0.15	−0.21	− 0.20	−0.15	1

*HFaST* Heart Failure Symptom Tracker

Note: HFaST values are as follows: 0 = Did not experience in the past 24 h, 1 = Much better than usual, 2 = Somewhat better than usual, 3 = Slightly better than usual, 4 = About the same as usual, 5 = Slightly worse than usual, 6 = Somewhat worse than usual, 7 = Much worse than usual. HFaST Item 10 values: 1 = yes, 2 = no

##### Test-retest reliability

Kappa statistics ranged from 0.71 to 0.95 across HFaST items 1 through 9, indicating substantial agreement in HFaST scores from one day to the next when patients’ symptoms were stable (i.e., corresponding PGISS change = 0) (Additional file 1: Table S8). Due to lack of variability (most patients [> 80%] reported no weight gain), kappa was not computed for “gaining more than 2 pounds in the past 24 hours or 5 pounds in the past 72 hours” (HFaST Item 10).

##### Construct validity and responsiveness

As anticipated, a greater proportion of patients who did not experience a particular symptom over the past 2 weeks based on the KCCQ-12 also reported that they did not experience the corresponding HFaST symptom (Table 3 for day 4). Most patients reporting no change on the PGISS also reported no change in HFaST symptoms from day to day (Table 4). Mean HFaST scores were lowest in the PGISS improvement category and higher in the PGISS worsening category, with median HFaST scores hovering around 4 (“about the same as usual”) in the PGISS no-change category.

**Table 3:** Construct Validity: HFaST Scores for Asymptomatic Patients Per the KCCQ-12 on Study Day 4

KCCQ-12: Symptom-free subgroup	HFaST Score	Did not experience in the past 24 h, n (%)	Much better than usual, n (%)	Somewhat better than usual, n (%)	Slightly better than usual, n (%)	About the same as usual, n (%)	Slightly worse than usual, n (%)	Somewhat worse than usual, n (%)	Much worse than usual, n (%)	Missing n (%)
No fatigue (Item 3), n (%)	HFaST 1 Fatigue or low energy level when performing everyday activities	10 (76.9)	1 (7.7)	0 (0)	1 (7.7)	1 (7.7)	0 (0)	0 (0)	0 (0)	0 (0.0)
	HFaST 2 Fatigue or low energy level even while sitting or lying down	11 (84.6)	1 (7.7)	0 (0)	1 (7.7)	0 (0)	0 (0)	0 (0)	0 (0)	0 (0.0)
No shortness of breath (Item 4), n (%)	HFaST 3 Shortness of breath when performing everyday activities	15 (75.0)	3 (15.0)	0 (0)	1 (5.0)	1 (5.0)	0 (0)	0 (0)	0 (0)	0 (0.0)
	HFaST 4 Shortness of breath at rest	16 (80.0)	2 (10.0)	1 (5.0)	1 (5.0)	0 (0)	0 (0)	0 (0)	0 (0)	0 (0.0)
No pillows (Item 5), n (%)	HFaST 5 Shortness of breath while lying down or reclining (for example, needing to add pillows or move to a recliner to sleep)	37 (72.5)	1 (2.0)	2 (3.9)	2 (3.9)	9 (17.6)	0 (0)	0 (0)	0 (0)	1 (1.9)
No swelling (Item 2), n (%)	HFaST 8 Swelling of feet, ankles, legs, or abdomen; shoes or waistband feeling tight	26 (78.8)	2 (6.1)	1 (3.0)	1 (3.0)	3 (9.1)	0 (0)	0 (0)	0 (0)	0 (0.0)

*HFaST* Heart Failure Symptom Tracker Questionnaire, *KCCQ-12* Kansas City Cardiomyopathy Questionnaire Short Form

**Table 4:** Construct Validity and Responsiveness: HFaST Scores by PGISS Categories

PGISS Item	HFaST Item on Day 5	PGISS Improvement from Day 4 to Day 5 Mean (SD), Median, Range, n	PGISS No Change from Day 4 to Day 5 Mean (SD), Median, Range, n	PGISS Worsening from Day 4 to Day 5 Mean (SD), Median, Range, n
PGISS 1 Fatigue	HFaST 1 Fatigue or low energy level when performing everyday activities	3.5 (1.2), 3.0, 2 to 5, n = 14	4.1 (1.1), 4.0, 1 to 7, n = 71	4.9 (1.1), 5.0, 4 to 7, n = 9
	HFaST 2 Fatigue or low energy level even while sitting or lying down	3.3 (1.0), 4.0, 1 to 4, *n* = 14	3.7 (1.5), 4.0, 1 to 7, *n* = 71	5.0 (1.2), 5.0, 4 to 7, *n* = 9
PGISS 2 Shortness of breath	HFaST 3 Shortness of breath when performing everyday activities	2.9 (1.4), 3.0, 1 to 5, n = 11	3.8 (1.3), 4.0, 1 to 7, n = 74	5.0 (0.6), 5.0, 4 to 6, n = 9
	HFaST 4 Shortness of breath at rest	1.8 (1.0), 1.5, 1 to 3, n = 11	3.4 (1.2), 4.0, 1 to 5, *n* = 74	3.9 (1.2), 4.0, 1 to 5, n = 9
	HFaST 5 Shortness of breath while lying down or reclining (for example, needing to add pillows or move to a recliner to sleep)	1.8 (1.0), 1.5, 1 to 3, *n* = 11	3.6 (1.1), 4.0, 1 to 6, *n* = 74	4.3 (0.8), 4.0, 3 to 5, n = 9
	HFaST 6 Sudden attacks of shortness of breath that wake you from sleeping	3.0 (1.7), 4.0, 1 to 4, n = 11	3.5 (1.5), 4.0, 1 to 6, n = 74	3.5 (1.7), 4.0, 1 to 5, n = 9
PGISS 3 Cough	HFaST 7 Cough	2.3 (1.3), 2.5, 1 to 4, *n* = 13	3.3 (1.4), 4.0, 1 to 5, *n* = 68	3.8 (1.6), 4.0, 1 to 6, *n* = 13
PGISS 4 Swelling	HFaST 8 Swelling of feet, ankles, legs, or abdomen; shoes or waistband feeling tight	3.2 (1.4), 4.0, 1 to 5, *n* = 14	3.6 (1.2), 4.0, 1 to 5, *n* = 72	5.0 (1.1), 5.0, 4 to 7, *n* = 8
PGISS 5 Heart palpitations	HFaST 9 Heart palpitations—rapid, fluttering, or pounding heartbeat	3.0 (1.7), 4.0, 1 to 4, n = 5	3.5 (1.4), 4.0, 1 to 7, *n* = 77	3.9 (1.6), 4.5, 1 to 5, *n* = 12

*HFaST* Heart Failure Symptom Tracker Questionnaire, *PGISS* Patient Global Impression of Symptom Severity, *SD* standard deviation

Note: HFaST values are as follows: 0 = Did not experience in the past 24 h, 1 = Much better than usual, 2 = Somewhat better than usual, 3 = Slightly better than usual, 4 = About the same as usual, 5 = Slightly worse than usual, 6 = Somewhat worse than usual, 7 = Much worse than usual

Table excludes patients who reported HFaST = “Did not experience” for PGISS Change From Day 4 to Day 5 and HFaST Day 5

##### Known-groups validity

As hypothesized, patients in the worsening group (based on the PGIC) exhibited statistically worse (higher) average HFaST scores than patients who improved or did not change (*P* < 0.05) for HFaST items 1 through 9 (Additional file 1: Table S9). “Gaining more than 2 pounds in the past 24 hours or 5 pounds in the past 72 hours” (HFaST Item 10) did not yield statistically significant known groups, likely on account of small cell sizes; very few patients indicated experiencing weight gain (from 6.3% on day 7 to 18.6% on day 4).

#### Feasibility and acceptability

HCPs rated the HFaST as a good (70%) or excellent (30%) means of assisting patients in keeping track of their HF symptoms and a good (80%) or excellent (20%) means of providing feedback to patients regarding their HF symptoms. All HCPs (100%) endorsed paper as the HFaST format most easily completed by patients, but most (70%) indicated that a web-based HFaST would be more easily utilized by HCPs.

**Fig. 2:**
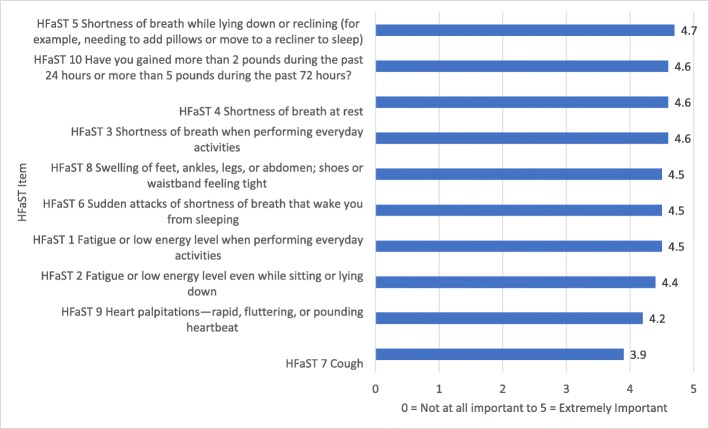
Draft HFaST Item Text and Ratings of Symptom Importance Per HCPs (*n* = 10)

HCPs reviewed the HFaST and provided feedback on the importance of each item (Fig. 2; Additional file 1: Table S10). On average, HCPs rated “shortness of breath while lying down or reclining” (HFaST Item 5) as the most important HFaST item (mean importance score, 4.7; SD, 0.7). “Cough” (HFaST Item 7) was endorsed as the least important item (mean importance score, 3.9; SD, 1.0).

#### Item reduction

Prevalence of an HF symptom (Additional file 1: Table S2) was not considered evidence for item reduction because patients experience their own set of HF symptoms depending on personal characteristics, NYHA classification, and other circumstances. For example, “gaining more than 2 pounds in the past 24 hours or 5 pounds in the past 72 hours” was not endorsed by most as a symptom they experienced during the study period, but this item was retained because, in both qualitative interviews and the HCP exit questionnaire, HCPs deemed this one of the most important symptoms to monitor.

**Fig. 3:**
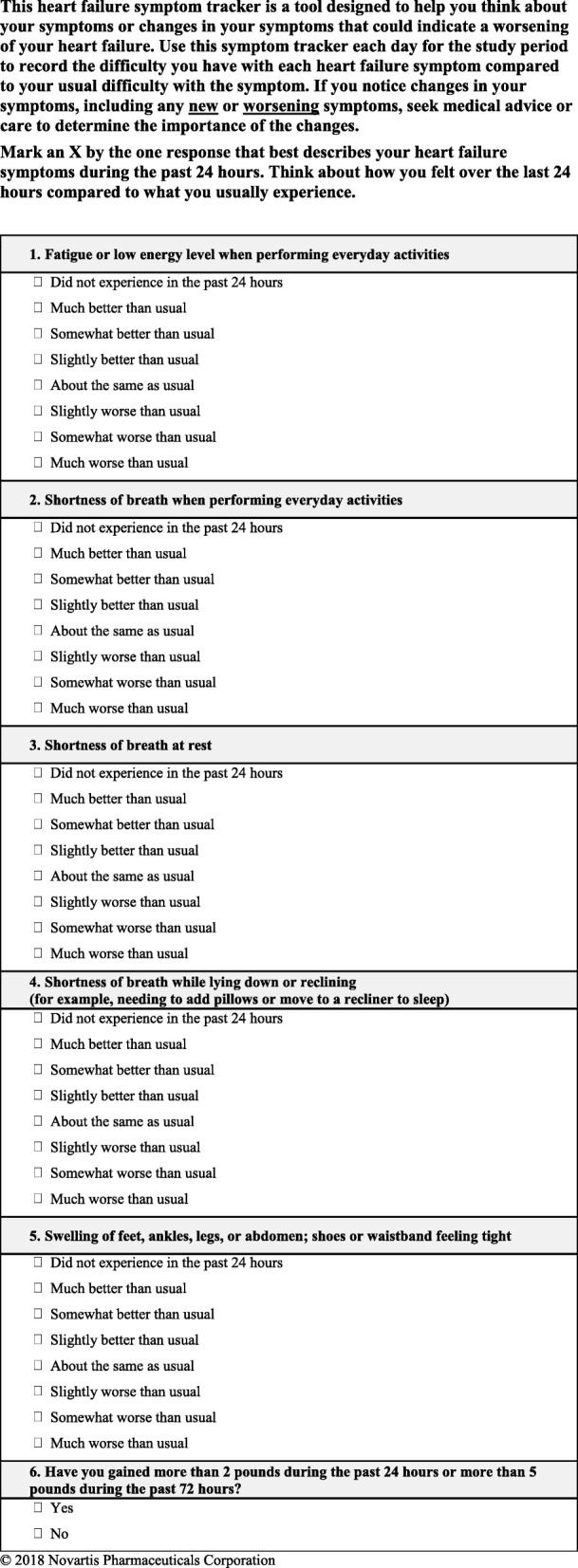
Final HFaST

To create a concise measure, only the most relevant and best-performing items were retained based on feedback from stakeholders in the qualitative study and results from the quantitative assessment. The following four draft items were removed, yielding the final 6-item communication tool (Fig 3):

- HFaST item 2: fatigue or low energy level even while sitting or lying down

The HFaST 2 was not highly ranked by stakeholders during qualitative interviews and exhibited strong inter-item correlations, suggesting an overlap of content with “fatigue or low energy level when performing everyday activities” (HFaST Item 1). Between these two items, HCPs rated HFaST Item 2 as less important than HFaST Item 1.

- HFaST item 6: sudden attacks of shortness of breath that wakes you from sleeping

Although highly ranked by patients and caregivers during qualitative interviews, HCPs did not endorse this concept as key. Additionally, the strong inter-item correlation with “shortness of breath while lying down or reclining” (HFaST Item 5) indicated redundancy. HCPs did not endorse HFaST Item 6 as one of the most important items to include.

- HFaST item 7: cough

Item 7 of the HFaST was endorsed by all stakeholders during qualitative interviews, and it showed acceptable test-retest reliability (kappa ≥0.90) and known-groups validity (*P* < 0.01). However, weaker inter-item correlations with other HFaST items (Table 2) suggest that cough may be a nonspecific symptom. HCPs ranked cough as the least important HF symptom in the exit survey.

- HFaST item 9: heart palpitations—rapid, fluttering, or pounding heartbeat

Health care providers ranked HFaST Item 9 as one of the least clinically relevant items indicating worsening HF symptoms.

## Discussion

Although existing measures are valuable for characterizing the severity and impact of HF, the HFaST was designed to facilitate patient-clinician communication on worsening HF symptoms. The HFaST addresses a gap identified in the current literature by addressing limitations of existing HF measures that focus on severity and impact of HF. Specifically, McHorney [20] recommended measures be brief and easy to administer, score, and interpret, but existing measures are longer and employ recall periods of 7 days or more [10]. All HF symptom measures identified by Lee and colleagues [10] included multiple domains, leading to more complicated scoring. Existing questionnaires that use a 7-day recall window are important for detecting general HF symptom severity or impact of the condition but cannot also measure precipitous changes requiring prompt medical attention. Psychometric evidence for existing measures is mixed, with some lacking important construct validity evidence [10]. The HFaST offers unique response choices to help patients identify *worsening* HF symptoms that require medical care. Although the HFaST can be administered via paper or web, HCPs indicated that patients would most likely prefer paper-based administration. One benefit of measuring change from usual rather than daily symptom severity is that patients and clinicians do not need to use scoring to calculate change (particularly important for paper-based administration), facilitating timely interpretation. Selection of a 24-h recall period was endorsed by stakeholders as the most accurate timeframe to report symptom changes. Additionally, this recall provides a flexible administration window (e.g., week, month) to support the anticipated variability in patient status (e.g., severity and stability of symptoms, newly diagnosed patients, recently discharged). Importantly, true operationalization of the tool will depend upon the particular situation faced by each patient as well as the utility in frequency of measurement anticipated by a treating physician and is an important part of future work.

The most relevant and best-performing items were identified and included in the final HFaST utilizing both qualitative and quantitative assessments. Psychometric evaluation provided initial evidence that the HFaST is reliable and valid as a communication tool for patients with HF over a 7-day period. Test-retest reliability was excellent for 5 of the 6 final HFaST items. Descriptive analyses supported construct validity and responsiveness: change in HFaST item responses was observed when change was expected based on external criteria. Known-groups analyses confirmed the discriminating ability of 5 of the 6 final HFaST items. Additionally, analyses utilizing the full item set may provide additional versions of the tool with utility across different contexts of use.

Some limitations should be acknowledged. Few patients experienced weight gain (HFaST Item 10) during the study period, and for some HFaST items, more than a third of patients did not experience the symptom being measured; thus, further research is recommended to confirm the measurement properties in a larger, more symptomatic sample. As a pilot study, the sample size was relatively small considering the prevalence of HF in the US, limiting generalizability. Although recruiting procedures attempted to ensure geographic and participant diversity, there was no formal sampling procedure, and use of a convenience sample could bias results. Approximately 60% of patients participated in paper-based administration, though it should be noted that two patient recruitment facilities provided *only* a web-based option. As such, it is unclear if patients prefer web- or paper-based administration, although HCPs indicated that patients would most likely prefer paper-based administration. However, electronic administration was not problematic to patients who took the HFaST as a web survey, and the ability to communicate results to HCPs electronically would facilitate rapid and frequent transmission of results.

In addition to daily administration, future studies should investigate different frequencies of administration to find a balance between patient burden (number of items) and quick identification of HFaST symptom changes. For example, less-stable patients (e.g., individuals who were more recently diagnosed, individuals who were recently discharged from the hospital) may benefit from daily HFaST administration, whereas patients with better controlled HF symptoms may require fewer weekly completions. Further, the HFaST instructions should be further evaluated for different frequencies of administration. Finally, the impact of implementing the HFaST in routine clinical care on patient outcomes should be assessed. Incorporating a longer data collection window (> 7 days) would allow researchers to obtain feedback from patients and HCPs on the feasibility of the HFaST in routine HF care. In addition, a longer data collection window would provide an opportunity to evaluate the efficacy of the HFaST as an intervention to potentially reduce hospitalization rates or identify other benefits arising from improved communication (e.g., patient satisfaction and engagement). Finally, future studies should develop a scoring algorithm and assess the validity of the HFaST to predict HF decompensation or uncontrolled symptoms indicative of the need to be seen by a clinician.

Curran and colleagues [21] propose methods to incorporate evaluations of clinical effectiveness and implementation research across three hybrid types. Hybrid type 1 includes testing effects of an intervention on relevant outcomes while observing and gathering information on implementation; type 2 includes a dual assessment and type 3, evaluation in conjunction with observation of the impact of the intervention of the outcome. Type 3 is most relevant to future HFaST assessment.

## Conclusions

The 6-item HFaST is a short, easy-to-use, and helpful communication tool designed for use in routine clinical care to raise patient awareness of new or worsening HF symptoms. Patient awareness of changing symptoms facilitates proactive communication with HCPs, maximizing the likelihood of timely intervention. Future studies should assess the efficacy of the HFaST as an intervention to increase patient-clinician communication and improve health outcomes (e.g., fewer hospitalizations, ER visits). In addition, future studies should evaluate implementation of HFaST in clinical practice and consider adopting a Hybrid Type 3 design to test an implementation strategy and collect information on the HFaST impact on prioritized outcomes (e.g., fewer hospitalizations, ER visits) [21]. A key strength of the HFaST is that it was developed with direct input from patients, caregivers, and HCPs and assesses patient perception of change in key HF symptoms from a usual state. Potential applications include monitoring patients with HF who were recently discharged from the hospital or tracking more stable HF patients to increase awareness of worsening symptoms that may require medical intervention. This pilot study provides initial support for the psychometric properties and feasibility of the HFaST as a communication tool in clinical practice.

## Additional file

Additional file 1: **Figure S1.** HCP Feasibility and Acceptability Questionnaire Topics. **Figure S2.** HFaST Initial 40 Items. **Table S1.** Summary Table of Heart Failure Symptom Tracker Item Evolution (40 to 20 items). NRS = numeric rating scale; RC = response choice; Y/N = yes/no. **Table S2.** HFaST Frequency Distributions by Study Day. **Table S3.** KCCQ Scale-Level Descriptive Statistics. **Table S4.** PGISS Item Descriptive Statistics by Study Day (*n* = 98). **Table S5.** Change in PGISS: Descriptive Statistics by Study Day. **Table S6.** Weight Descriptive Statistics by Study Day. **Table S7.** HFaST Inter-Item Correlations (*n* = 91 to 98). **Table S8.** Test-Retest Kappa Statistics: HFaST Item-Level Scores. **Table S9.** Known-Groups ANOVAs: PGIC. **Table S10.** Ratings of Symptom Importance to Be Included in the HFaST: Per Clinicians. (DOCX 325 kb)

## Abbreviations

ER: Emergency room
HCP: Health care providers
HF: Heart failure
HFaST: HF Symptom Tracker
KCCQ-12: Kansas City Cardiomyopathy Questionnaire
NYHA: New York Heart Association
PGIC: Patient global impression of change
PGISS: Patient global impression of symptom severity
PRO: Patient-reported outcome
SD: Standard deviation
US: United States
