# Supplementary material for: Development, psychometric evaluation, and initial feasibility assessment of a symptom tracker for use by patients with heart failure (HFaST)
Source: J Patient Rep Outcomes. 2019 May 2;3:26. doi: 10.1186/s41687-019-0113-6 (PMC6497706; doi:10.1186/s41687-019-0113-6)
Supplement: Supplementary file 1 — Figure S1. HCP Feasibility and Acceptability Questionnaire Topics. Figure S2. HFaST Initial 40 Items. Table S1. Summary Table of Heart Failure Symptom Tracker Item Evolution (40 to 20 items). NRS = numeric rating scale; RC = response choice; Y/N = yes/no. Table S2. HFaST Frequency Distributions by Study Day. Table S3. KCCQ Scale-Level Descriptive Statistics. Table S4. PGISS Item Descriptive Statistics by Study Day (n = 98). Table S5. Change in PGISS: Descriptive Statistics by Study Day. Table S6. Weight Descriptive Statistics by Study Day. Table S7. HFaST Inter-Item Correlations (n = 91 to 98). Table S8. Test-Retest Kappa Statistics: HFaST Item-Level Scores. Table S9. Known-Groups ANOVAs: PGIC. Table S10. Ratings of Symptom Importance to Be Included in the HFaST: Per Clinicians. (DOCX 325 kb) [file 41687_2019_113_MOESM1_ESM.docx]

SUPPLEMENTAL MATERIALS

Figure S-1. HCP Feasibility and Acceptability Questionnaire Topics

^a^ Type of practice: solo, group, university-based, hospital-based, other.

^b^ Cardiology, surgery, internal medicine, family practice, other.

Figure S-2. HFaST Initial 40 Items


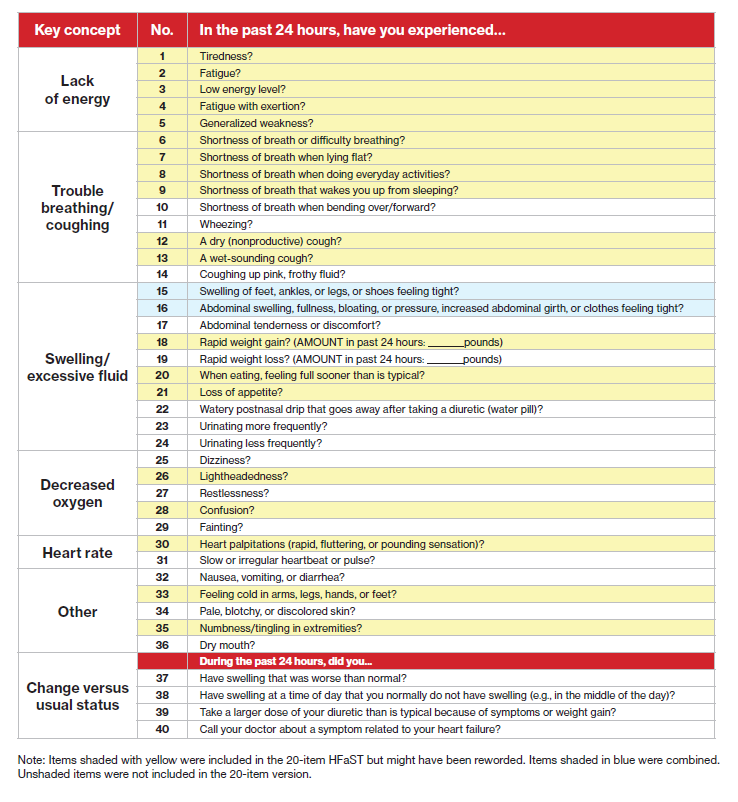


1. Summary Table of Heart Failure Symptom Tracker Item Evolution (40 to 20 items)

| 40-Item Version | 20-Item Version |
| --- | --- |
| RC: 5-pt scale, none – very severe | RC: 11-point NRS with 2 anchors |
| Instructions: Please record the severity of any symptom you have experienced in the past 24 hours or select “none” if you have not experienced the symptom. | Instructions: Circle the one number that describes how much difficulty you have had with each of the following symptoms during the past 24 hours, with 0 meaning you have not had the symptom and 10 meaning your difficulty with that symptom was the worst that you could imagine. |
| Tiredness? | Tiredness? |
| Fatigue? | Fatigue? |
| Low energy level? | Low energy level? |
| Fatigue with exertion? | Low energy level when performing everyday activities? |
| Generalized weakness? | Generalized weakness? |
| Shortness of breath or difficulty breathing? | Increased shortness of breath or difficulty breathing? |
| Shortness of breath when doing everyday activities? | Increased shortness of breath when doing everyday activities? |
| Shortness of breath when lying flat? | Shortness of breath that requires use of additional pillows or upright position when sleeping? |
| Shortness of breath that wakes you up from sleeping? | Shortness of breath that wakes you up from sleeping? |
| Shortness of breath when bending over/forward? |  |
| Wheezing? |  |
| A dry (nonproductive) cough? | A dry (nonproductive) cough? |
| A wet-sounding cough? | A wet-sounding cough? |
| Coughing up pink, frothy fluid? |  |
| Swelling of feet, ankles, or legs, or shoes feeling tight? | Swelling of abdomen, feet, ankles, or legs, or shoes or waistband feeling tight? |
| Abdominal swelling, fullness, bloating, or pressure, increased abdominal girth, or clothes feeling tight? |  |
| Abdominal tenderness or discomfort? |  |
| Rapid weight gain?  (AMOUNT in past 24 hours: _______pounds) | Rapid weight gain?  (AMOUNT in past 24 hours: _______pounds) |
| Rapid weight loss?  (AMOUNT in past 24 hours: _______pounds) |  |
| When eating, feeling full sooner than is typical? | When eating, feeling full sooner than is typical? |
| Loss of appetite? | Loss of appetite? |
| Watery postnasal drip that goes away after taking a diuretic (water pill)? |  |
| Urinating more frequently? |  |
| Urinating less frequently? |  |
| Dizziness? | Dizziness or lightheadedness? |
| Lightheadedness? |  |
| Restlessness? |  |
| Confusion? | Feeling confused (things are not making sense)? |
| Fainting? |  |
| Heart palpitations (rapid, fluttering, or pounding sensation)? | Rapid, fluttering, or pounding heartbeat? |
| Slow or irregular heartbeat or pulse? |  |
| Nausea, vomiting, or diarrhea? |  |
| Feeling cold in arms, legs, hands, or feet? | Feeling cold in arms, legs, hands, or feet? |
| Pale, blotchy, or discolored skin? |  |
| Numbness/tingling in extremities? | Numbness or tingling in hands or feet? |
| Dry mouth? |  |
| **CHANGE VS. USUAL**  **During the past 24 hours, did you….** |  |
| Have swelling that was worse than normal? (Y/N) |  |
| Have swelling at a time of day that you normally do not have swelling (e.g., in the middle of the day)? (Y/N) |  |
| Take a larger dose of your diuretic than is typical because of symptoms or weight gain? (Y/N) |  |
| Call your doctor about a symptom related to your heart failure? (Y/N) |  |

1. NRS = numeric rating scale; RC = response choice; Y/N = yes/noHFaST Frequency Distributions by Study Day

| HFaST Score | Day | Did not experience in the past 24 hours* | Much better than usual* | Somewhat better than usual* | Slightly better than usual* | About the same as usual* | Slightly worse than usual* | Somewhat worse than usual* | Much worse than usual* | Missing (%) |
| --- | --- | --- | --- | --- | --- | --- | --- | --- | --- | --- |
| HFaST 1 Fatigue or low energy level when performing everyday activities |  |  |  |  |  |  |  |  |  |  |
|  | 1 | 12 (12.2) | 9 (9.2) | 4 (4.1) | 7 (7.1) | 40 (40.8) | 18 (18.4) | 7 (7.1) | 1 (1.0) | 0 (0.0) |
|  | 2 | 18 (19.4) | 9 (9.7) | 8 (8.6) | 12 (12.9) | 32 (34.4) | 11 (11.8) | 2 (2.2) | 1 (1.1) | 5 (5.1) |
|  | 3 | 19 (19.8) | 9 (9.4) | 5 (5.2) | 9 (9.4) | 26 (27.1) | 22 (22.9) | 6 (6.3) | 0 (0) | 2 (2.0) |
|  | 4 | 22 (22.9) | 3 (3.1) | 8 (8.3) | 8 (8.3) | 34 (35.4) | 16 (16.7) | 4 (4.2) | 1 (1.0) | 2 (2.0) |
|  | 5 | 23 (24.0) | 1 (1.0) | 8 (8.3) | 9 (9.4) | 26 (27.1) | 25 (26.0) | 2 (2.1) | 2 (2.1) | 2 (2.0) |
|  | 6 | 22 (22.9) | 7 (7.3) | 11 (11.5) | 6 (6.3) | 22 (22.9) | 21 (21.9) | 4 (4.2) | 3 (3.1) | 2 (2.0) |
|  | 7 | 23 (24.0) | 8 (8.3) | 5 (5.2) | 8 (8.3) | 25 (26.0) | 21 (21.9) | 5 (5.2) | 1 (1.0) | 2 (2.0) |
| HFaST 2 Fatigue or low energy level even while sitting or lying down |  |  |  |  |  |  |  |  |  |  |
|  | 1 | 21 (21.6) | 7 (7.2) | 7 (7.2) | 9 (9.3) | 35 (36.1) | 16 (16.5) | 2 (2.1) | 0 (0) | 1 (1.0) |
|  | 2 | 27 (29.0) | 6 (6.5) | 9 (9.7) | 8 (8.6) | 36 (38.7) | 4 (4.3) | 3 (3.2) | 0 (0) | 5 (5.1) |
|  | 3 | 29 (30.2) | 7 (7.3) | 6 (6.3) | 3 (3.1) | 35 (36.5) | 13 (13.5) | 3 (3.1) | 0 (0) | 2 (2.0) |
|  | 4 | 32 (33.7) | 5 (5.3) | 1 (1.1) | 10 (10.5) | 29 (30.5) | 13 (13.7) | 5 (5.3) | 0 (0) | 3 (3.1) |
|  | 5 | 31 (33.3) | 6 (6.5) | 5 (5.4) | 8 (8.6) | 26 (28.0) | 12 (12.9) | 3 (3.2) | 2 (2.2) | 5 (5.1) |
|  | 6 | 32 (34.0) | 11 (11.7) | 6 (6.4) | 5 (5.3) | 21 (22.3) | 13 (13.8) | 5 (5.3) | 1 (1.1) | 4 (4.1) |
|  | 7 | 30 (32.6) | 6 (6.5) | 4 (4.3) | 14 (15.2) | 24 (26.1) | 9 (9.8) | 3 (3.3) | 2 (2.2) | 6 (6.1) |
| HFaST 3 Shortness of breath when performing everyday activities |  |  |  |  |  |  |  |  |  |  |
|  | 1 | 19 (19.6) | 11 (11.3) | 5 (5.2) | 9 (9.3) | 23 (23.7) | 23 (23.7) | 6 (6.2) | 1 (1.0) | 1 (1.0) |
|  | 2 | 24 (25.8) | 9 (9.7) | 6 (6.5) | 4 (4.3) | 31 (33.3) | 15 (16.1) | 3 (3.2) | 1 (1.1) | 5 (5.1) |
|  | 3 | 23 (24.0) | 9 (9.4) | 5 (5.2) | 6 (6.3) | 25 (26.0) | 23 (24.0) | 4 (4.2) | 1 (1.0) | 2 (2.0) |
|  | 4 | 31 (32.6) | 3 (3.2) | 7 (7.4) | 5 (5.3) | 29 (30.5) | 16 (16.8) | 4 (4.2) | 0 (0) | 3 (3.1) |
|  | 5 | 27 (29.3) | 5 (5.4) | 7 (7.6) | 8 (8.7) | 25 (27.2) | 17 (18.5) | 2 (2.2) | 1 (1.1) | 6 (6.1) |
|  | 6 | 26 (27.4) | 5 (5.3) | 7 (7.4) | 4 (4.2) | 24 (25.3) | 22 (23.2) | 3 (3.2) | 4 (4.2) | 3 (3.1) |
|  | 7 | 29 (31.2) | 7 (7.5) | 4 (4.3) | 9 (9.7) | 21 (22.6) | 17 (18.3) | 4 (4.3) | 2 (2.2) | 5 (5.1) |
| HFaST 4 Shortness of breath at rest |  |  |  |  |  |  |  |  |  |  |
|  | 1 | 38 (40.0) | 6 (6.3) | 4 (4.2) | 2 (2.1) | 34 (35.8) | 10 (10.5) | 0 (0) | 1 (1.1) | 3 (3.1) |
|  | 2 | 42 (44.7) | 6 (6.4) | 3 (3.2) | 13 (13.8) | 26 (27.7) | 3 (3.2) | 1 (1.1) | 0 (0) | 4 (4.1) |
|  | 3 | 39 (41.1) | 8 (8.4) | 3 (3.2) | 7 (7.4) | 27 (28.4) | 9 (9.5) | 2 (2.1) | 0 (0) | 3 (3.1) |
|  | 4 | 41 (42.3) | 5 (5.2) | 5 (5.2) | 10 (10.3) | 27 (27.8) | 9 (9.3) | 0 (0) | 0 (0) | 1 (1.0) |
|  | 5 | 42 (43.8) | 9 (9.4) | 5 (5.2) | 7 (7.3) | 27 (28.1) | 6 (6.3) | 0 (0) | 0 (0) | 2 (2.0) |
|  | 6 | 43 (45.3) | 6 (6.3) | 3 (3.2) | 10 (10.5) | 18 (18.9) | 11 (11.6) | 3 (3.2) | 1 (1.1) | 3 (3.1) |
|  | 7 | 44 (45.8) | 6 (6.3) | 3 (3.1) | 8 (8.3) | 20 (20.8) | 14 (14.6) | 1 (1.0) | 0 (0) | 2 (2.0) |
| HFaST 5 Shortness of breath while lying down or reclining (for example, needing to add pillows or move to a recliner to sleep) |  |  |  |  |  |  |  |  |  |  |
|  | 1 | 38 (40.0) | 2 (2.1) | 5 (5.3) | 6 (6.3) | 33 (34.7) | 7 (7.4) | 4 (4.2) | 0 (0) | 3 (3.1) |
|  | 2 | 51 (54.3) | 2 (2.1) | 4 (4.3) | 2 (2.1) | 24 (25.5) | 9 (9.6) | 1 (1.1) | 1 (1.1) | 4 (4.1) |
|  | 3 | 46 (48.4) | 4 (4.2) | 4 (4.2) | 4 (4.2) | 24 (25.3) | 12 (12.6) | 1 (1.1) | 0 (0) | 3 (3.1) |
|  | 4 | 51 (52.6) | 3 (3.1) | 5 (5.2) | 5 (5.2) | 28 (28.9) | 4 (4.1) | 0 (0) | 1 (1.0) | 1 (1.0) |
|  | 5 | 50 (52.1) | 3 (3.1) | 7 (7.3) | 7 (7.3) | 22 (22.9) | 6 (6.3) | 1 (1.0) | 0 (0) | 2 (2.0) |
|  | 6 | 51 (53.7) | 4 (4.2) | 4 (4.2) | 6 (6.3) | 18 (18.9) | 8 (8.4) | 3 (3.2) | 1 (1.1) | 3 (3.1) |
|  | 7 | 55 (57.3) | 4 (4.2) | 5 (5.2) | 5 (5.2) | 15 (15.6) | 11 (11.5) | 1 (1.0) | 0 (0) | 2 (2.0) |
| HFaST 6 Sudden attacks of shortness of breath that wake you from sleeping |  |  |  |  |  |  |  |  |  |  |
|  | 1 | 57 (58.2) | 9 (9.2) | 1 (1.0) | 8 (8.2) | 14 (14.3) | 7 (7.1) | 2 (2.0) | 0 (0) | 0 (0.0) |
|  | 2 | 63 (67.0) | 7 (7.4) | 4 (4.3) | 2 (2.1) | 12 (12.8) | 5 (5.3) | 1 (1.1) | 0 (0) | 4 (4.1) |
|  | 3 | 62 (64.6) | 6 (6.3) | 2 (2.1) | 3 (3.1) | 16 (16.7) | 4 (4.2) | 3 (3.1) | 0 (0) | 2 (2.0) |
|  | 4 | 64 (68.1) | 4 (4.3) | 4 (4.3) | 4 (4.3) | 12 (12.8) | 6 (6.4) | 0 (0) | 0 (0) | 4 (4.1) |
|  | 5 | 64 (67.4) | 7 (7.4) | 1 (1.1) | 1 (1.1) | 16 (16.8) | 5 (5.3) | 1 (1.1) | 0 (0) | 3 (3.1) |
|  | 6 | 64 (66.7) | 6 (6.3) | 2 (2.1) | 2 (2.1) | 9 (9.4) | 7 (7.3) | 6 (6.3) | 0 (0) | 2 (2.0) |
|  | 7 | 65 (68.4) | 5 (5.3) | 1 (1.1) | 3 (3.2) | 11 (11.6) | 6 (6.3) | 3 (3.2) | 1 (1.1) | 3 (3.1) |
| HFaST 7 Cough |  |  |  |  |  |  |  |  |  |  |
|  | 1 | 33 (34.0) | 9 (9.3) | 6 (6.2) | 7 (7.2) | 25 (25.8) | 10 (10.3) | 6 (6.2) | 1 (1.0) | 1 (1.0) |
|  | 2 | 35 (37.2) | 9 (9.6) | 6 (6.4) | 8 (8.5) | 27 (28.7) | 7 (7.4) | 1 (1.1) | 1 (1.1) | 4 (4.1) |
|  | 3 | 36 (37.5) | 9 (9.4) | 5 (5.2) | 10 (10.4) | 19 (19.8) | 16 (16.7) | 1 (1.0) | 0 (0) | 2 (2.0) |
|  | 4 | 41 (42.7) | 8 (8.3) | 7 (7.3) | 10 (10.4) | 23 (24.0) | 7 (7.3) | 0 (0) | 0 (0) | 2 (2.0) |
|  | 5 | 41 (43.2) | 10 (10.5) | 8 (8.4) | 8 (8.4) | 17 (17.9) | 10 (10.5) | 1 (1.1) | 0 (0) | 3 (3.1) |
|  | 6 | 42 (43.8) | 9 (9.4) | 4 (4.2) | 12 (12.5) | 16 (16.7) | 10 (10.4) | 3 (3.1) | 0 (0) | 2 (2.0) |
|  | 7 | 42 (44.2) | 10 (10.5) | 2 (2.1) | 7 (7.4) | 20 (21.1) | 12 (12.6) | 1 (1.1) | 1 (1.1) | 3 (3.1) |
| HFaST 8 Swelling of feet, ankles, legs, or abdomen; shoes or waistband feeling tight |  |  |  |  |  |  |  |  |  |  |
|  | 1 | 39 (39.8) | 6 (6.1) | 5 (5.1) | 6 (6.1) | 23 (23.5) | 15 (15.3) | 2 (2.0) | 2 (2.0) | 0 (0.0) |
|  | 2 | 36 (38.7) | 3 (3.2) | 7 (7.5) | 11 (11.8) | 26 (28.0) | 9 (9.7) | 0 (0) | 1 (1.1) | 5 (5.1) |
|  | 3 | 37 (38.5) | 8 (8.3) | 5 (5.2) | 7 (7.3) | 25 (26.0) | 9 (9.4) | 3 (3.1) | 2 (2.1) | 2 (2.0) |
|  | 4 | 41 (42.3) | 6 (6.2) | 5 (5.2) | 8 (8.2) | 23 (23.7) | 10 (10.3) | 3 (3.1) | 1 (1.0) | 1 (1.0) |
|  | 5 | 40 (42.6) | 6 (6.4) | 4 (4.3) | 6 (6.4) | 26 (27.7) | 11 (11.7) | 0 (0) | 1 (1.1) | 4 (4.1) |
|  | 6 | 40 (42.1) | 8 (8.4) | 5 (5.3) | 8 (8.4) | 21 (22.1) | 10 (10.5) | 3 (3.2) | 0 (0) | 3 (3.1) |
|  | 7 | 41 (42.7) | 10 (10.4) | 5 (5.2) | 6 (6.3) | 21 (21.9) | 12 (12.5) | 1 (1.0) | 0 (0) | 2 (2.0) |
| HFaST 9 Heart palpitations—rapid, fluttering, or pounding heartbeat |  |  |  |  |  |  |  |  |  |  |
|  | 1 | 55 (56.1) | 6 (6.1) | 5 (5.1) | 3 (3.1) | 21 (21.4) | 5 (5.1) | 2 (2.0) | 1 (1.0) | 0 (0.0) |
|  | 2 | 56 (60.2) | 4 (4.3) | 2 (2.2) | 5 (5.4) | 20 (21.5) | 5 (5.4) | 1 (1.1) | 0 (0) | 5 (5.1) |
|  | 3 | 56 (58.9) | 7 (7.4) | 2 (2.1) | 5 (5.3) | 15 (15.8) | 8 (8.4) | 0 (0) | 2 (2.1) | 3 (3.1) |
|  | 4 | 59 (60.8) | 4 (4.1) | 2 (2.1) | 2 (2.1) | 21 (21.6) | 7 (7.2) | 1 (1.0) | 1 (1.0) | 1 (1.0) |
|  | 5 | 54 (58.1) | 7 (7.5) | 1 (1.1) | 4 (4.3) | 20 (21.5) | 5 (5.4) | 1 (1.1) | 1 (1.1) | 5 (5.1) |
|  | 6 | 52 (54.7) | 5 (5.3) | 0 (0) | 5 (5.3) | 20 (21.1) | 10 (10.5) | 1 (1.1) | 2 (2.1) | 3 (3.1) |
|  | 7 | 54 (56.8) | 3 (3.2) | 1 (1.1) | 3 (3.2) | 23 (24.2) | 10 (10.5) | 1 (1.1) | 0 (0) | 3 (3.1) |
| HFaST 10 Gained more than 2 pounds during the past 24 hours or more than 5 pounds during the past 72 hours |  | No | Yes |  |  |  |  |  |  |  |
|  | 1 | 84 (85.7) | 14 (14.3) |  |  |  |  |  |  | 0 (0.0) |
|  | 2 | 88 (93.6) | 6 (6.4) |  |  |  |  |  |  | 4 (4.1) |
|  | 3 | 84 (88.4) | 11 (11.6) |  |  |  |  |  |  | 3 (3.1) |
|  | 4 | 79 (81.4) | 18 (18.6) |  |  |  |  |  |  | 1 (1.0) |
|  | 5 | 83 (87.4) | 12 (12.6) |  |  |  |  |  |  | 3 (3.1) |
|  | 6 | 83 (87.4) | 12 (12.6) |  |  |  |  |  |  | 3 (3.1) |
|  | 7 | 89 (93.7) | 6 (6.3) |  |  |  |  |  |  | 3 (3.1) |

HFaST = Heart Failure Symptom Tracker; min = minimum; max = maximum; SD = standard deviation.

Note: HFaST values are as follows: 0 = Did not experience in the past 24 hours, 1 = Much better than usual, 2 = Somewhat better than usual, 3 = Slightly better than usual, 4 = About the same as usual, 5 = Slightly worse than usual, 6 = Somewhat worse than usual, 7 = Much worse than usual.

* Percent calculated out of non-missing responses.

1. KCCQ Scale-Level Descriptive Statistics

| KCCQ-12 Scores | n | Mean (SD) | Median | Min to Max | Missing (%) |
| --- | --- | --- | --- | --- | --- |
| KCCQ Physical Limitation Score | 89 | 53.2 (25.7) | 50.0 | 0 to 100 | 9 (9.2%) |
| KCCQ Symptom Frequency Score | 94 | 58.8 (26.9) | 59.4 | 0 to 100 | 4 (4.1%) |
| KCCQ Quality of Life Score | 92 | 46.6 (27.5) | 50.0 | 0 to 100 | 6 (6.1%) |
| KCCQ Social Limitation score | 90 | 52.6 (28.3) | 50.0 | 0 to 100 | 8 (8.2%) |
| KCCQ Summary score | 94 | 53.1 (24.3) | 53.1 | 4 to 100 | 4 (4.1%) |

KCCQ-12 = Kansas City Cardiomyopathy Questionnaire; min = minimum; max = maximum SD = standard deviation.

1. PGISS Item Descriptive Statistics by Study Day (n = 98)

| PGISS Score | Day | None | Mild | Moderate | Severe | Very Severe | Missing (%) | Mean (SD), median; min-max, n |
| --- | --- | --- | --- | --- | --- | --- | --- | --- |
| PGISS 1 Fatigue |  |  |  |  |  |  |  |  |
|  | 1 | 20 (20.4) | 34 (34.7) | 39 (39.8) | 5 (5.1) | 0 (0) | 0 (0.0) | 1.30 (0.9), 1.0; 0-3, 98 |
|  | 2 | 20 (21.3) | 28 (29.8) | 41 (43.6) | 5 (5.3) | 0 (0) | 4 (4.1) | 1.33 (0.9), 1.0; 0-3, 94 |
|  | 3 | 16 (16.8) | 40 (42.1) | 30 (31.6) | 9 (9.5) | 0 (0) | 3 (3.1) | 1.34 (0.9), 1.0; 0-3, 95 |
|  | 4 | 23 (23.7) | 34 (35.1) | 34 (35.1) | 6 (6.2) | 0 (0) | 1 (1.0) | 1.24 (0.9), 1.0; 0-3, 97 |
|  | 5 | 21 (22.1) | 36 (37.9) | 37 (38.9) | 1 (1.1) | 0 (0) | 3 (3.1) | 1.19 (0.8), 1.0; 0-3, 95 |
|  | 6 | 23 (24.2) | 35 (36.8) | 28 (29.5) | 9 (9.5) | 0 (0) | 3 (3.1) | 1.24 (0.9), 1.0; 0-3, 95 |
|  | 7 | 23 (24.2) | 38 (40.0) | 26 (27.4) | 7 (7.4) | 1 (1.1) | 3 (3.1) | 1.21 (0.9), 1.0; 0-4, 95 |
| PGISS 2 Shortness of breath |  |  |  |  |  |  |  |  |
|  | 1 | 26 (26.5) | 36 (36.7) | 29 (29.6) | 6 (6.1) | 1 (1.0) | 0 (0.0) | 1.18 (0.9), 1.0; 0-4, 98 |
|  | 2 | 28 (29.8) | 27 (28.7) | 34 (36.2) | 5 (5.3) | 0 (0) | 4 (4.1) | 1.17 (0.9), 1.0; 0-3, 94 |
|  | 3 | 27 (28.1) | 33 (34.4) | 29 (30.2) | 7 (7.3) | 0 (0) | 2 (2.0) | 1.17 (0.9), 1.0; 0-3, 96 |
|  | 4 | 30 (30.9) | 35 (36.1) | 29 (29.9) | 3 (3.1) | 0 (0) | 1 (1.0) | 1.05 (0.9), 1.0; 0-3, 97 |
|  | 5 | 30 (31.6) | 36 (37.9) | 25 (26.3) | 4 (4.2) | 0 (0) | 3 (3.1) | 1.03 (0.9), 1.0; 0-3, 95 |
|  | 6 | 31 (33.0) | 32 (34.0) | 24 (25.5) | 7 (7.4) | 0 (0) | 4 (4.1) | 1.07 (0.9), 1.0; 0-3, 94 |
|  | 7 | 31 (32.6) | 35 (36.8) | 19 (20.0) | 9 (9.5) | 1 (1.1) | 3 (3.1) | 1.09 (1.0), 1.0; 0-4, 95 |
| PGISS 3 Cough |  |  |  |  |  |  |  |  |
|  | 1 | 39 (39.8) | 35 (35.7) | 19 (19.4) | 5 (5.1) | 0 (0) | 0 (0.0) | 0.90 (0.9), 1.0; 0-3, 98 |
|  | 2 | 42 (44.7) | 30 (31.9) | 19 (20.2) | 3 (3.2) | 0 (0) | 4 (4.1) | 0.82 (0.9), 1.0; 0-3, 94 |
|  | 3 | 39 (40.6) | 32 (33.3) | 22 (22.9) | 3 (3.1) | 0 (0) | 2 (2.0) | 0.89 (0.9), 1.0; 0-3, 96 |
|  | 4 | 45 (46.4) | 32 (33.0) | 18 (18.6) | 2 (2.1) | 0 (0) | 1 (1.0) | 0.76 (0.8), 1.0; 0-3, 97 |
|  | 5 | 46 (48.4) | 26 (27.4) | 20 (21.1) | 3 (3.2) | 0 (0) | 3 (3.1) | 0.79 (0.9), 1.0; 0-3, 95 |
|  | 6 | 44 (46.3) | 29 (30.5) | 15 (15.8) | 6 (6.3) | 1 (1.1) | 3 (3.1) | 0.85 (1.0), 1.0; 0-4, 95 |
|  | 7 | 43 (45.3) | 33 (34.7) | 15 (15.8) | 3 (3.2) | 1 (1.1) | 3 (3.1) | 0.80 (0.9), 1.0; 0-4, 95 |
| PGISS 4 Swelling |  |  |  |  |  |  |  |  |
|  | 1 | 42 (42.9) | 30 (30.6) | 20 (20.4) | 6 (6.1) | 0 (0) | 0 (0.0) | 0.90 (0.9), 1.0; 0-3, 98 |
|  | 2 | 39 (41.5) | 33 (35.1) | 19 (20.2) | 3 (3.2) | 0 (0) | 4 (4.1) | 0.85 (0.9), 1.0; 0-3, 94 |
|  | 3 | 41 (42.7) | 28 (29.2) | 21 (21.9) | 6 (6.3) | 0 (0) | 2 (2.0) | 0.92 (0.9), 1.0; 0-3, 96 |
|  | 4 | 44 (45.4) | 30 (30.9) | 21 (21.6) | 2 (2.1) | 0 (0) | 1 (1.0) | 0.80 (0.8), 1.0; 0-3, 97 |
|  | 5 | 41 (43.2) | 38 (40.0) | 14 (14.7) | 1 (1.1) | 1 (1.1) | 3 (3.1) | 0.77 (0.8), 1.0; 0-4, 95 |
|  | 6 | 44 (46.3) | 32 (33.7) | 14 (14.7) | 5 (5.3) | 0 (0) | 3 (3.1) | 0.79 (0.9), 1.0; 0-3, 95 |
|  | 7 | 42 (44.2) | 31 (32.6) | 20 (21.1) | 2 (2.1) | 0 (0) | 3 (3.1) | 0.81 (0.8), 1.0; 0-3, 95 |
| PGISS 5 Heart palpitations |  |  |  |  |  |  |  |  |
|  | 1 | 59 (60.2) | 23 (23.5) | 12 (12.2) | 4 (4.1) | 0 (0) | 0 (0.0) | 0.60 (0.9), 0.0; 0-3, 98 |
|  | 2 | 57 (60.6) | 19 (20.2) | 17 (18.1) | 1 (1.1) | 0 (0) | 4 (4.1) | 0.60 (0.8), 0.0; 0-3, 94 |
|  | 3 | 57 (59.4) | 20 (20.8) | 16 (16.7) | 3 (3.1) | 0 (0) | 2 (2.0) | 0.64 (0.9), 0.0; 0-3, 96 |
|  | 4 | 60 (61.9) | 17 (17.5) | 18 (18.6) | 2 (2.1) | 0 (0) | 1 (1.0) | 0.61 (0.9), 0.0; 0-3, 97 |
|  | 5 | 56 (58.9) | 16 (16.8) | 21 (22.1) | 2 (2.1) | 0 (0) | 3 (3.1) | 0.67 (0.9), 0.0; 0-3, 95 |
|  | 6 | 52 (54.7) | 21 (22.1) | 16 (16.8) | 6 (6.3) | 0 (0) | 3 (3.1) | 0.75 (1.0), 0.0; 0-3, 95 |
|  | 7 | 54 (56.8) | 21 (22.1) | 15 (15.8) | 5 (5.3) | 0 (0) | 3 (3.1) | 0.69 (0.9), 0.0; 0-3, 95 |

PGISS = Patient Global Impression of Symptom Severity; min = minimum; max = maximum; SD = standard deviation.

Note: Percent calculated out of non-missing responses.

1. Change in PGISS: Descriptive Statistics by Study Day

| PGISS Score | Day | n | Mean (SD) | Median | Min to Max | Missing (%) |
| --- | --- | --- | --- | --- | --- | --- |
| PGISS 1 Fatigue |  |  |  |  |  |  |
|  | Day 2 - Day 1 | 94 | 0.0 (0.6) | 0.0 | –2 to 2 | 4 (4.1%) |
|  | Day 3 - Day 2 | 91 | –0.0 (0.6) | 0.0 | –1 to 1 | 7 (7.1%) |
|  | Day 4 - Day 3 | 94 | –0.1 (0.6) | 0.0 | –2 to 2 | 4 (4.1%) |
|  | Day 5 - Day 4 | 94 | –0.1 (0.5) | 0.0 | –1 to 1 | 4 (4.1%) |
|  | Day 6 - Day 5 | 92 | 0.1 (0.6) | 0.0 | –1 to 2 | 6 (6.1%) |
|  | Day 7 - Day 6 | 92 | –0.1 (0.6) | 0.0 | –2 to 2 | 6 (6.1%) |
| PGISS 2 Shortness of breath |  |  |  |  |  |  |
|  | Day 2 - Day 1 | 94 | –0.0 (0.6) | 0.0 | –2 to 1 | 4 (4.1%) |
|  | Day 3 - Day 2 | 92 | –0.0 (0.6) | 0.0 | –2 to 1 | 6 (6.1%) |
|  | Day 4 - Day 3 | 95 | –0.1 (0.6) | 0.0 | –2 to 2 | 3 (3.1%) |
|  | Day 5 - Day 4 | 94 | –0.0 (0.5) | 0.0 | –2 to 2 | 4 (4.1%) |
|  | Day 6 - Day 5 | 91 | 0.1 (0.5) | 0.0 | –2 to 1 | 7 (7.1%) |
|  | Day 7 - Day 6 | 91 | 0.0 (0.7) | 0.0 | –2 to 2 | 7 (7.1%) |
| PGISS 3 Cough |  |  |  |  |  |  |
|  | Day 2 - Day 1 | 94 | –0.1 (0.7) | 0.0 | –2 to 2 | 4 (4.1%) |
|  | Day 3 - Day 2 | 92 | 0.1 (0.6) | 0.0 | –1 to 2 | 6 (6.1%) |
|  | Day 4 - Day 3 | 95 | –0.1 (0.6) | 0.0 | –2 to 1 | 3 (3.1%) |
|  | Day 5 - Day 4 | 94 | 0.0 (0.6) | 0.0 | –1 to 2 | 4 (4.1%) |
|  | Day 6 - Day 5 | 92 | 0.1 (0.6) | 0.0 | –2 to 2 | 6 (6.1%) |
|  | Day 7 - Day 6 | 92 | –0.0 (0.7) | 0.0 | –2 to 2 | 6 (6.1%) |
| PGISS 4 Swelling |  |  |  |  |  |  |
|  | Day 2 - Day 1 | 94 | –0.1 (0.7) | 0.0 | –3 to 1 | 4 (4.1%) |
|  | Day 3 - Day 2 | 92 | 0.1 (0.6) | 0.0 | –1 to 3 | 6 (6.1%) |
|  | Day 4 - Day 3 | 95 | –0.1 (0.7) | 0.0 | –3 to 2 | 3 (3.1%) |
|  | Day 5 - Day 4 | 94 | –0.1 (0.5) | 0.0 | –1 to 2 | 4 (4.1%) |
|  | Day 6 - Day 5 | 92 | 0.0 (0.7) | 0.0 | –2 to 2 | 6 (6.1%) |
|  | Day 7 - Day 6 | 92 | 0.0 (0.6) | 0.0 | –1 to 2 | 6 (6.1%) |
| PGISS 5 Heart palpitations |  |  |  |  |  |  |
|  | Day 2 - Day 1 | 94 | –0.0 (0.6) | 0.0 | –2 to 2 | 4 (4.1%) |
|  | Day 3 - Day 2 | 92 | 0.0 (0.6) | 0.0 | –1 to 2 | 6 (6.1%) |
|  | Day 4 - Day 3 | 95 | –0.0 (0.5) | 0.0 | –2 to 1 | 3 (3.1%) |
|  | Day 5 - Day 4 | 94 | 0.1 (0.4) | 0.0 | –1 to 1 | 4 (4.1%) |
|  | Day 6 - Day 5 | 92 | 0.1 (0.5) | 0.0 | –2 to 2 | 6 (6.1%) |
|  | Day 7 - Day 6 | 92 | –0.0 (0.5) | 0.0 | –1 to 3 | 6 (6.1%) |

PGISS = Patient Global Impression of Symptom Severity; min = minimum; max = maximum; SD = standard deviation.

Note: PGISS values range from 0 = none to 4 = very severe and change in PGISS ranges from -4 (improved) to 4 (worsening).

1. Weight Descriptive Statistics by Study Day

| Weight | n | Mean (SD) | Median | Min to Max | Missing (%) |
| --- | --- | --- | --- | --- | --- |
| Day 1 | 95 | 221.5 (58.8) | 223.0 | 118 to 426 | 3 (3.1%) |
| Day 2 | 94 | 220.7 (57.2) | 223.0 | 118 to 426 | 4 (4.1%) |
| Day 3 | 96 | 222.4 (57.7) | 221.5 | 125 to 426 | 2 (2.0%) |
| Day 4 | 95 | 221.2 (59.4) | 221.0 | 117 to 426 | 3 (3.1%) |
| Day 5 | 94 | 220.9 (58.9) | 220.9 | 118 to 426 | 4 (4.1%) |
| Day 6 | 96 | 220.8 (58.7) | 222.4 | 118 to 426 | 2 (2.0%) |
| Day 7 | 95 | 220.9 (58.6) | 221.2 | 115 to 426 | 3 (3.1%) |

1. HFaST Inter-Item Correlations (n = 91 to 98)

| HFaST Item | 1 | 2 | 3 | 4 | 5 | 6 | 7 | 8 | 9 | 10 |
| --- | --- | --- | --- | --- | --- | --- | --- | --- | --- | --- |
| Day 1 |  |  |  |  |  |  |  |  |  |  |
| HFaST 1 Fatigue or low energy level when performing everyday activities | 1 |  |  |  |  |  |  |  |  |  |
| HFaST 2 Fatigue or low energy level even while sitting or lying down | 0.74 | 1 |  |  |  |  |  |  |  |  |
| HFaST 3 Shortness of breath when performing everyday activities | 0.65 | 0.56 | 1 |  |  |  |  |  |  |  |
| HFaST 4 Shortness of breath at rest | 0.52 | 0.69 | 0.77 | 1 |  |  |  |  |  |  |
| HFaST 5 Shortness of breath while lying down or reclining (for example, needing to add pillows or move to a recliner to sleep) | 0.56 | 0.70 | 0.73 | 0.89 | 1 |  |  |  |  |  |
| HFaST 6 Sudden attacks of shortness of breath that wake you from sleeping | 0.32 | 0.47 | 0.56 | 0.69 | 0.74 | 1 |  |  |  |  |
| HFaST 7 Cough | 0.21 | 0.14 | 0.30 | 0.34 | 0.36 | 0.33 | 1 |  |  |  |
| HFaST 8 Swelling of feet, ankles, legs, or abdomen; shoes or waistband feeling tight | 0.46 | 0.61 | 0.55 | 0.56 | 0.53 | 0.49 | 0.43 | 1 |  |  |
| HFaST 9 Heart palpitations—rapid, fluttering, or pounding heartbeat | 0.49 | 0.51 | 0.39 | 0.38 | 0.41 | 0.54 | 0.21 | 0.46 | 1 |  |
| HFaST 10 Have you gained more than 2 pounds during the past 24 hours or more than 5 pounds during the past 72 hours? | –0.09 | –0.35 | –0.11 | –0.19 | –0.17 | –0.35 | –0.22 | –0.45 | –0.24 | 1 |
| Day 2 |  |  |  |  |  |  |  |  |  |  |
| HFaST 1 Fatigue or low energy level when performing everyday activities | 1 |  |  |  |  |  |  |  |  |  |
| HFaST 2 Fatigue or low energy level even while sitting or lying down | 0.76 | 1 |  |  |  |  |  |  |  |  |
| HFaST 3 Shortness of breath when performing everyday activities | 0.68 | 0.60 | 1 |  |  |  |  |  |  |  |
| HFaST 4 Shortness of breath at rest | 0.58 | 0.63 | 0.69 | 1 |  |  |  |  |  |  |
| HFaST 5 Shortness of breath while lying down or reclining (for example, needing to add pillows or move to a recliner to sleep) | 0.60 | 0.61 | 0.66 | 0.88 | 1 |  |  |  |  |  |
| HFaST 6 Sudden attacks of shortness of breath that wake you from sleeping | 0.55 | 0.55 | 0.51 | 0.65 | 0.76 | 1 |  |  |  |  |
| HFaST 7 Cough | 0.33 | 0.48 | 0.33 | 0.49 | 0.47 | 0.50 | 1 |  |  |  |
| HFaST 8 Swelling of feet, ankles, legs, or abdomen; shoes or waistband feeling tight | 0.50 | 0.68 | 0.51 | 0.57 | 0.56 | 0.37 | 0.39 | 1 |  |  |
| HFaST 9 Heart palpitations—rapid, fluttering, or pounding heartbeat | 0.49 | 0.59 | 0.49 | 0.65 | 0.64 | 0.62 | 0.43 | 0.42 | 1 |  |
| HFaST 10 Have you gained more than 2 pounds during the past 24 hours or more than 5 pounds during the past 72 hours? | –0.53 | –0.29 | –0.27 | –0.22 | –0.15 | –0.61 | –0.25 | –0.47 | –0.47 | 1 |
| Day 3 |  |  |  |  |  |  |  |  |  |  |
| HFaST 1 Fatigue or low energy level when performing everyday activities | 1 |  |  |  |  |  |  |  |  |  |
| HFaST 2 Fatigue or low energy level even while sitting or lying down | 0.86 | 1 |  |  |  |  |  |  |  |  |
| HFaST 3 Shortness of breath when performing everyday activities | 0.77 | 0.70 | 1 |  |  |  |  |  |  |  |
| HFaST 4 Shortness of breath at rest | 0.57 | 0.65 | 0.82 | 1 |  |  |  |  |  |  |
| HFaST 5 Shortness of breath while lying down or reclining (for example, needing to add pillows or move to a recliner to sleep) | 0.62 | 0.67 | 0.75 | 0.84 | 1 |  |  |  |  |  |
| HFaST 6 Sudden attacks of shortness of breath that wake you from sleeping | 0.55 | 0.63 | 0.60 | 0.79 | 0.86 | 1 |  |  |  |  |
| HFaST 7 Cough | 0.44 | 0.35 | 0.62 | 0.45 | 0.40 | 0.44 | 1 |  |  |  |
| HFaST 8 Swelling of feet, ankles, legs, or abdomen; shoes or waistband feeling tight | 0.56 | 0.61 | 0.66 | 0.62 | 0.65 | 0.55 | 0.38 | 1 |  |  |
| HFaST 9 Heart palpitations—rapid, fluttering, or pounding heartbeat | 0.69 | 0.71 | 0.74 | 0.51 | 0.59 | 0.51 | 0.51 | 0.58 | 1 |  |
| HFaST 10 Have you gained more than 2 pounds during the past 24 hours or more than 5 pounds during the past 72 hours? | –0.08 | –0.09 | –0.19 | –0.13 | –0.22 | –0.00 | –0.27 | –0.51 | –0.17 | 1 |
| Day 4 |  |  |  |  |  |  |  |  |  |  |
| HFaST 1 Fatigue or low energy level when performing everyday activities | 1 |  |  |  |  |  |  |  |  |  |
| HFaST 2 Fatigue or low energy level even while sitting or lying down | 0.82 | 1 |  |  |  |  |  |  |  |  |
| HFaST 3 Shortness of breath when performing everyday activities | 0.62 | 0.67 | 1 |  |  |  |  |  |  |  |
| HFaST 4 Shortness of breath at rest | 0.53 | 0.68 | 0.75 | 1 |  |  |  |  |  |  |
| HFaST 5 Shortness of breath while lying down or reclining (for example, needing to add pillows or move to a recliner to sleep) | 0.56 | 0.68 | 0.74 | 0.91 | 1 |  |  |  |  |  |
| HFaST 6 Sudden attacks of shortness of breath that wake you from sleeping | 0.45 | 0.49 | 0.52 | 0.74 | 0.81 | 1 |  |  |  |  |
| HFaST 7 Cough | 0.46 | 0.33 | 0.56 | 0.58 | 0.57 | 0.44 | 1 |  |  |  |
| HFaST 8 Swelling of feet, ankles, legs, or abdomen; shoes or waistband feeling tight | 0.62 | 0.48 | 0.53 | 0.60 | 0.60 | 0.56 | 0.51 | 1 |  |  |
| HFaST 9 Heart palpitations—rapid, fluttering, or pounding heartbeat | 0.54 | 0.69 | 0.64 | 0.67 | 0.69 | 0.53 | 0.53 | 0.45 | 1 |  |
| HFaST 10 Have you gained more than 2 pounds during the past 24 hours or more than 5 pounds during the past 72 hours? | –0.05 | –0.05 | –0.27 | –0.12 | –0.13 | –0.15 | –0.21 | –0.20 | –0.15 | 1 |
| Day 5 |  |  |  |  |  |  |  |  |  |  |
| HFaST 1 Fatigue or low energy level when performing everyday activities | 1 |  |  |  |  |  |  |  |  |  |
| HFaST 2 Fatigue or low energy level even while sitting or lying down | 0.83 | 1 |  |  |  |  |  |  |  |  |
| HFaST 3 Shortness of breath when performing everyday activities | 0.78 | 0.74 | 1 |  |  |  |  |  |  |  |
| HFaST 4 Shortness of breath at rest | 0.61 | 0.82 | 0.67 | 1 |  |  |  |  |  |  |
| HFaST 5 Shortness of breath while lying down or reclining (for example, needing to add pillows or move to a recliner to sleep) | 0.65 | 0.76 | 0.74 | 0.89 | 1 |  |  |  |  |  |
| HFaST 6 Sudden attacks of shortness of breath that wake you from sleeping | 0.41 | 0.50 | 0.64 | 0.71 | 0.84 | 1 |  |  |  |  |
| HFaST 7 Cough | 0.30 | 0.38 | 0.47 | 0.48 | 0.38 | 0.41 | 1 |  |  |  |
| HFaST 8 Swelling of feet, ankles, legs, or abdomen; shoes or waistband feeling tight | 0.53 | 0.54 | 0.58 | 0.45 | 0.40 | 0.24 | 0.37 | 1 |  |  |
| HFaST 9 Heart palpitations—rapid, fluttering, or pounding heartbeat | 0.61 | 0.66 | 0.68 | 0.72 | 0.69 | 0.55 | 0.53 | 0.48 | 1 |  |
| HFaST 10 Have you gained more than 2 pounds during the past 24 hours or more than 5 pounds during the past 72 hours? | –0.25 | –0.29 | –0.21 | –0.33 | –0.18 | –0.16 | 0.05 | –0.39 | –0.16 | 1 |
| Day 6 |  |  |  |  |  |  |  |  |  |  |
| HFaST 1 Fatigue or low energy level when performing everyday activities | 1 |  |  |  |  |  |  |  |  |  |
| HFaST 2 Fatigue or low energy level even while sitting or lying down | 0.88 | 1 |  |  |  |  |  |  |  |  |
| HFaST 3 Shortness of breath when performing everyday activities | 0.88 | 0.75 | 1 |  |  |  |  |  |  |  |
| HFaST 4 Shortness of breath at rest | 0.81 | 0.89 | 0.84 | 1 |  |  |  |  |  |  |
| HFaST 5 Shortness of breath while lying down or reclining (for example, needing to add pillows or move to a recliner to sleep) | 0.79 | 0.79 | 0.78 | 0.86 | 1 |  |  |  |  |  |
| HFaST 6 Sudden attacks of shortness of breath that wake you from sleeping | 0.63 | 0.72 | 0.61 | 0.81 | 0.80 | 1 |  |  |  |  |
| HFaST 7 Cough | 0.45 | 0.20 | 0.49 | 0.38 | 0.51 | 0.38 | 1 |  |  |  |
| HFaST 8 Swelling of feet, ankles, legs, or abdomen; shoes or waistband feeling tight | 0.44 | 0.54 | 0.48 | 0.53 | 0.44 | 0.36 | 0.48 | 1 |  |  |
| HFaST 9 Heart palpitations—rapid, fluttering, or pounding heartbeat | 0.66 | 0.55 | 0.72 | 0.70 | 0.57 | 0.56 | 0.48 | 0.39 | 1 |  |
| HFaST 10 Have you gained more than 2 pounds during the past 24 hours or more than 5 pounds during the past 72 hours? | –0.41 | –0.42 | –0.52 | –0.43 | –0.44 | –0.31 | –0.30 | –0.47 | –0.39 | 1 |
| **Day 7** |  |  |  |  |  |  |  |  |  |  |
| HFaST 1 Fatigue or low energy level when performing everyday activities | 1 |  |  |  |  |  |  |  |  |  |
| HFaST 2 Fatigue or low energy level even while sitting or lying down | 0.90 | 1 |  |  |  |  |  |  |  |  |
| HFaST 3 Shortness of breath when performing everyday activities | 0.83 | 0.79 | 1 |  |  |  |  |  |  |  |
| HFaST 4 Shortness of breath at rest | 0.76 | 0.87 | 0.85 | 1 |  |  |  |  |  |  |
| HFaST 5 Shortness of breath while lying down or reclining (for example, needing to add pillows or move to a recliner to sleep) | 0.75 | 0.78 | 0.78 | 0.88 | 1 |  |  |  |  |  |
| HFaST 6 Sudden attacks of shortness of breath that wake you from sleeping | 0.70 | 0.71 | 0.74 | 0.82 | 0.92 | 1 |  |  |  |  |
| HFaST 7 Cough | 0.38 | 0.32 | 0.46 | 0.38 | 0.52 | 0.51 | 1 |  |  |  |
| HFaST 8 Swelling of feet, ankles, legs, or abdomen; shoes or waistband feeling tight | 0.54 | 0.57 | 0.67 | 0.49 | 0.37 | 0.37 | 0.33 | 1 |  |  |
| HFaST 9 Heart palpitations—rapid, fluttering, or pounding heartbeat | 0.68 | 0.70 | 0.66 | 0.64 | 0.75 | 0.68 | 0.49 | 0.43 | 1 |  |
| HFaST 10 Have you gained more than 2 pounds during the past 24 hours or more than 5 pounds during the past 72 hours? | –0.22 | –0.33 | –0.35 | –0.47 | –0.41 | –0.47 | 0.04 | –0.28 | –0.25 | 1 |

HFaST = Heart Failure Symptom Tracker.

Note: HFaST values are as follows: 0 = Did not experience in the past 24 hours, 1 = Much better than usual, 2 = Somewhat better than usual, 3 = Slightly better than usual, 4 = About the same as usual, 5 = Slightly worse than usual, 6 = Somewhat worse than usual, 7 = Much worse than usual. HFaST item 10 values: 1 = yes, 2 = no.

1. Test-Retest Kappa Statistics: HFaST Item-Level Scores

|  | **Kappa (95% CI), n** | | | | |
| --- | --- | --- | --- | --- | --- |
| No Change in PGISS for 3 Consecutive Days | HFaST Day 2 compared to HFaST Day 3* | HFaST Day 3 compared to HFaST Day 4^†^ | HFaST Day 4 compared to HFaST Day 5^‡^ | HFaST Day 5 compared to HFaST Day^§^ | HFaST Day 6 compared to HFaST Day 7^\| \|^ |
| No change in PGISS 1 Fatigue |  |  |  |  |  |
| HFaST 1 Fatigue or low energy level when performing everyday activities | 0.82 (0.67-0.98), 46 | 0.92 (0.87-0.98), 50 | 0.87 (0.78-0.95), 53 | 0.88 (0.80-0.97), 52 | 0.88 (0.77-0.98), 56 |
| HFaST 2 Fatigue or low energy level even while sitting or lying down | 0.77 (0.61-0.93), 45 | 0.87 (0.74-1.00), 48 | 0.78 (0.61-0.94), 49 | 0.87 (0.74-0.99), 49 | 0.86 (0.74-0.97), 55 |
| No change in PGISS 2 Shortness of breath |  |  |  |  |  |
| HFaST 3 Shortness of breath when performing everyday activities | 0.80 (0.66-0.93), 47 | 0.90 (0.80-1.00), 52 | 0.89 (0.78-1.00), 53 | 0.83 (0.71-0.95), 53 | 0.92 (0.85-0.98), 46 |
| HFaST 4 Shortness of breath at rest | 0.79 (0.66-0.92), 47 | 0.79 (0.65-0.93), 52 | 0.81 (0.66-0.95), 57 | 0.74 (0.58-0.90), 58 | 0.82 (0.65-0.99), 48 |
| HFaST 5 Shortness of breath while lying down or reclining (for example, needing to add pillows or move to a recliner to sleep) | 0.72 (0.53-0.91), 48 | 0.88 (0.76-0.99), 53 | 0.75 (0.58-0.91), 57 | 0.75 (0.59-0.91), 58 | 0.84 (0.68-0.99), 48 |
| HFaST 6 Sudden attacks of shortness of breath that wake you from sleeping | 0.75 (0.53-0.96), 48 | 0.85 (0.71-1.00), 50 | 0.82 (0.66-0.98), 53 | 0.82 (0.69-0.96), 57 | 0.79 (0.59-0.99), 47 |
| No change in PGISS 3 Cough |  |  |  |  |  |
| HFaST 7 Cough | 0.90 (0.80-1.00), 45 | 0.90 (0.80-1.00), 53 | 0.95 (0.92-0.99), 51 | 0.94 (0.88-1.00), 53 | 0.90 (0.81-0.99), 59 |
| No change in in PGISS 4 Swelling |  |  |  |  |  |
| HFaST 8 Swelling of feet, ankles, legs, or abdomen; shoes or waistband feeling tight | 0.95 (0.91-0.99), 55 | 0.94 (0.90-0.98), 60 | 0.87 (0.78-0.96), 57 | 0.90 (0.83-0.98), 51 | 0.87 (0.75-0.99), 50 |
| HFaST 10 Have you gained more than 2 pounds during the past 24 hours or more than 5 pounds during the past 72 hours?^¶^ | — | — | — | — | — |
| No change in PGISS 5 Heart palpitations |  |  |  |  |  |
| HFaST 9 Heart palpitations—rapid, fluttering, or pounding heartbeat | 0.85 (0.73-0.98), 58 | 0.71 (0.46-0.95), 59 | 0.85 (0.72-0.99), 58 | 0.95 (0.89-1.00), 59 | 0.97 (0.94-0.99), 61 |

CI = confidence interval; HFaST = Heart Failure Symptom Tracker Questionnaire.

* No change in PGISS from day 1 through day 3.

^†^ No change in PGISS from day 2 through day 4.

^‡^ No change in PGISS from day 3 through day 5.

^§^ No change in PGISS from day 4 through day 6.

^| |^ No change in PGISS from day 5 through day 7.

^¶^ Kappa results could not be interpreted because of restricted range.

1. Known-Groups ANOVAs: PGIC

|  | PGIC Improvement or No Change (PGIC = 1, 2, 3, or 4) | | PGIC Worsening (PGIC = 5, 6, 7) | |  |
| --- | --- | --- | --- | --- | --- |
| Mean HFaST Score Day 1-Day 7 | n | Mean (SD) | n | Mean (SD) | F-statistic (*p*-value) |
| HFaST 1 Fatigue or low energy level when performing everyday activities | 82 | 2.9 (1.7) | 13 | 4.5 (0.9) | 11.40 (0.001) |
| HFaST 2 Fatigue or low energy level even while sitting or lying down | 82 | 2.4 (1.8) | 13 | 4.1 (0.9) | 12.59 (0.001) |
| HFaST 3 Shortness of breath when performing everyday activities | 82 | 2.6 (1.8) | 13 | 4.5 (0.9) | 14.13 (0.000) |
| HFaST 4 Shortness of breath at rest | 82 | 1.8 (1.7) | 13 | 3.3 (1.6) | 8.85 (0.004) |
| HFaST 5 Shortness of breath while lying down or reclining (for example, needing to add pillows or move to a recliner to sleep) | 82 | 1.6 (1.6) | 13 | 3.5 (1.6) | 16.37 (0.000) |
| HFaST 6 Sudden attacks of shortness of breath that wake you from sleeping | 82 | 1.0 (1.4) | 13 | 2.8 (2.1) | 17.01 (0.000) |
| HFaST 7 Cough | 82 | 1.8 (1.6) | 13 | 3.2 (1.9) | 7.95 (0.006) |
| HFaST 8 Swelling of feet, ankles, legs, or abdomen; shoes or waistband feeling tight | 82 | 2.0 (1.8) | 13 | 3.2 (2.0) | 5.03 (0.027) |
| HFaST 9 Heart palpitations—rapid, fluttering, or pounding heartbeat | 82 | 1.3 (1.6) | 13 | 3.0 (1.8) | 12.40 (0.001) |
| HFaST 10 Gained more than 2 pounds during the past 24 hours or more than 5 pounds during the past 72 hours | 82 | 1.9 (0.2) | 13 | 1.9 (0.2) | 0.06 (0.807) |

ANOVA = analysis of variance; SD = standard deviation; HFaST = Heart Failure Symptom Tracker Questionnaire; PGIC = Patient Global Impression of Change.

Note: HFaST values are as follows: 0 = Did not experience in the past 24 hours, 1 = Much better than usual, 2 = Somewhat better than usual, 3 = Slightly better than usual, 4 = About the same as usual, 5 = Slightly worse than usual, 6 = Somewhat worse than usual, 7 = Much worse than usual. HFaST item 10 values: 1 = yes, 2 = no.

1. Ratings of Symptom Importance to Be Included in the HFaST: Per Clinicians

| Symptom | Not at all important N (%) | Slightly important N (%) | Moderately important N (%) | Very important N (%) | Extremely important N (%) | Missing (%) | Mean (SD), median; min-max, n |
| --- | --- | --- | --- | --- | --- | --- | --- |
| HFaST 1 Fatigue or low energy level when performing everyday activities | 0 (0) | 0 (0) | 0 (0) | 5 (50.0) | 5 (50.0) | 0 (0.0) | 4.50 (0.5), 4.5; 4-5, 10 |
| HFaST 2 Fatigue or low energy level even while sitting or lying down | 0 (0) | 0 (0) | 1 (10.0) | 4 (40.0) | 5 (50.0) | 0 (0.0) | 4.40 (0.7), 4.5; 3-5, 10 |
| HFaST 3 Shortness of breath when performing everyday activities | 0 (0) | 0 (0) | 0 (0) | 4 (40.0) | 6 (60.0) | 0 (0.0) | 4.60 (0.5), 5.0; 4-5, 10 |
| HFaST 4 Shortness of breath at rest | 0 (0) | 0 (0) | 1 (10.0) | 2 (20.0) | 7 (70.0) | 0 (0.0) | 4.60 (0.7), 5.0; 3-5, 10 |
| HFaST 5 Shortness of breath while lying down or reclining (for example, needing to add pillows or move to a recliner to sleep) | 0 (0) | 0 (0) | 1 (10.0) | 1 (10.0) | 8 (80.0) | 0 (0.0) | 4.70 (0.7), 5.0; 3-5, 10 |
| HFaST 6 Sudden attacks of shortness of breath that wake you from sleeping | 0 (0) | 1 (10.0) | 0 (0) | 2 (20.0) | 7 (70.0) | 0 (0.0) | 4.50 (1.0), 5.0; 2-5, 10 |
| HFaST 7 Cough | 0 (0) | 1 (10.0) | 2 (20.0) | 4 (40.0) | 3 (30.0) | 0 (0.0) | 3.90 (1.0), 4.0; 2-5, 10 |
| HFaST 8 Swelling of feet, ankles, legs, or abdomen; shoes or waistband feeling tight | 0 (0) | 0 (0) | 0 (0) | 5 (50.0) | 5 (50.0) | 0 (0.0) | 4.50 (0.5), 4.5; 4-5, 10 |
| HFaST 9 Heart palpitations—rapid, fluttering, or pounding heartbeat | 0 (0) | 0 (0) | 2 (20.0) | 4 (40.0) | 4 (40.0) | 0 (0.0) | 4.20 (0.8), 4.0; 3-5, 10 |
| HFaST 10 Gained more than 2 pounds during the past 24 hours or more than 5 pounds during the past 72 hours | 0 (0) | 0 (0) | 0 (0) | 4 (40.0) | 6 (60.0) | 0 (0.0) | 4.60 (0.5), 5.0; 4-5, 10 |

Note: Percent calculated out of non-missing responses.
